# Formation of Heteropolycyclic Frameworks via the Dearomatization of a Dihapto-Coordinated Anisole

**DOI:** 10.1021/acs.organomet.5c00410

**Published:** 2025-11-20

**Authors:** Mason R. Ortiz, Justin T. Weatherford-Pratt, Jeremy M. Bloch, Diane A. Dickie, W. Dean Harman

**Affiliations:** Department of Chemistry, 2358 University of Virginia, Charlottesville, Virginia 22904, United States

## Abstract

Heteropolycyclic frameworks are widely represented in biologically and pharmaceutically relevant compounds; however, methods to synthesize these frameworks often result in heterocycles containing predominantly sp^2^-hybridized carbons. Herein we describe a heteroannulation scheme featuring a double protonation of a tungsten η^2^-anisole complex. The resulting dicationic intermediate reacts with activated arenes through an electrophilic aromatic substitution reaction to form an oxocarbenium complex, which can be reduced to an allylic ether complex. Subsequent acidolysis results in a π-allyl complex that can react with alcohol or amine substituents of the activated arene reagent to form the desired heteropolycyclic core.

## Graphical abstract

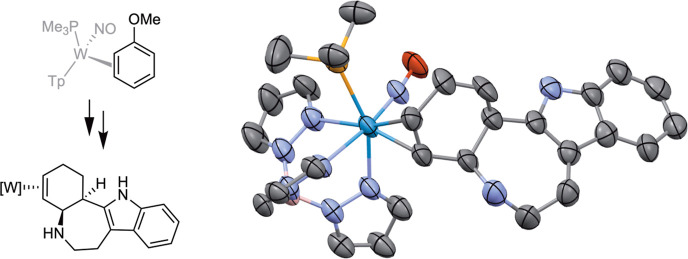

## Introduction

Owing to the high prevalence of heteropolycyclics in natural products, pharmaceuticals, and agrochemicals,^1^ new methods for accessing these compounds are of considerable interest to synthetic chemists. Many of these heteropolycyclics are considered “privileged scaffolds,” capable of interacting with multiple biological targets with high affinity.^2^ A well-established strategy for forming heteropolycyclic cores is through palladium-catalyzed heteroannulation reactions, which typically involve an oxidative addition of an aryl, vinylic, or allylic halide to Pd(0) followed by an insertion/reductive elimination sequence with an arene, alkene, or alkyne.^3^ The resulting compounds are typically “flat” in that the newly formed heterocyclic ring is made up of sp^2^-hybridized carbons.^4^ In this regard, mixtures of stereoisomers are avoided, but the compounds formed tend to lack sufficient three-dimensional complexity to selectively interact with receptor sites of target proteins. In contrast, most naturally occurring, biologically active compounds are rich in carbon stereocenters and are optimized for specific target receptors and minimal off-target binding. Since more complex molecules are better able to optimally fill the space of the receptor binding site, molecular complexity is strongly correlated with clinical success.^4^

Over the past three decades, our research group has developed the chemistry of dihapto-coordinated (η^2^) arene complexes of osmium,^5^ rhenium,^6^ molybdenum,^7^ and tungsten.^7^ With the highly π-basic metal bound to only two carbons of the aromatic ring, the remaining four carbons are available for the addition of various chemical fragments that, if bridged together, could create heteropolycyclic structures concomitant with the formation of new stereocenters.^7^ With the appropriate choices of commercially available “bridges,” new families of heteropolycyclic structures could be produced that could find use in diversity-oriented synthesis.^8–10^ In this investigation, we aim to demonstrate the potential of an η^2^-coordinated anisole complex to serve as the scaffold for new heteropolycyclic frameworks.

When a π-basic transition metal complex binds to an arene in an η^2^-fashion, electron density flows from the metal into the arene π-system. Correspondingly, early examples of the synthesis of polycyclic compounds from η^2^-arenes were initiated by addition of carbon electrophiles (Figure 1). These include Michael acceptors (**A**, **B**, **D–F**),^11–13^ acetals (**C**),^11^ ketenes (**G**),^14^ and carbenes (**H**).^15^ The goal of our most recent study was to develop complementary methods for the formation of polycyclic cores using nucleophilic bridges.^16^ Whereas that study exploited the electron-withdrawing properties of a sulfonyl group, our current investigation leverages the π-basic nature of a tungsten–anisole complex. Specifically, treating complex **1** with strong acid enables a double-protonation of the anisole ligand to give the dicationic intermediate (Figure 1; **2**).^17^ This highly electrophilic species reacts with electron-rich aromatic nucleophiles (e.g., phenols, anisoles, indoles) via an electrophilic aromatic substitution (EAS) reaction.^17,18^ Reduction of the resulting oxocarbenium complex (Figure 1, **I**) followed by acidolysis of the allylic ether (**II**) generates a π-allyl complex (**III**). We reasoned that with the use of a suitable aromatic tied to a heteroatom nucleophile (Ar–XH), heterocyclic scaffolds could be accessed through an intramolecular EAS/nucleophilic addition sequence (**I** → **IV**; Figure 1) without the need for precious-metal catalysts or specialized arenes (e.g., halogenated).

**Figure 1:**
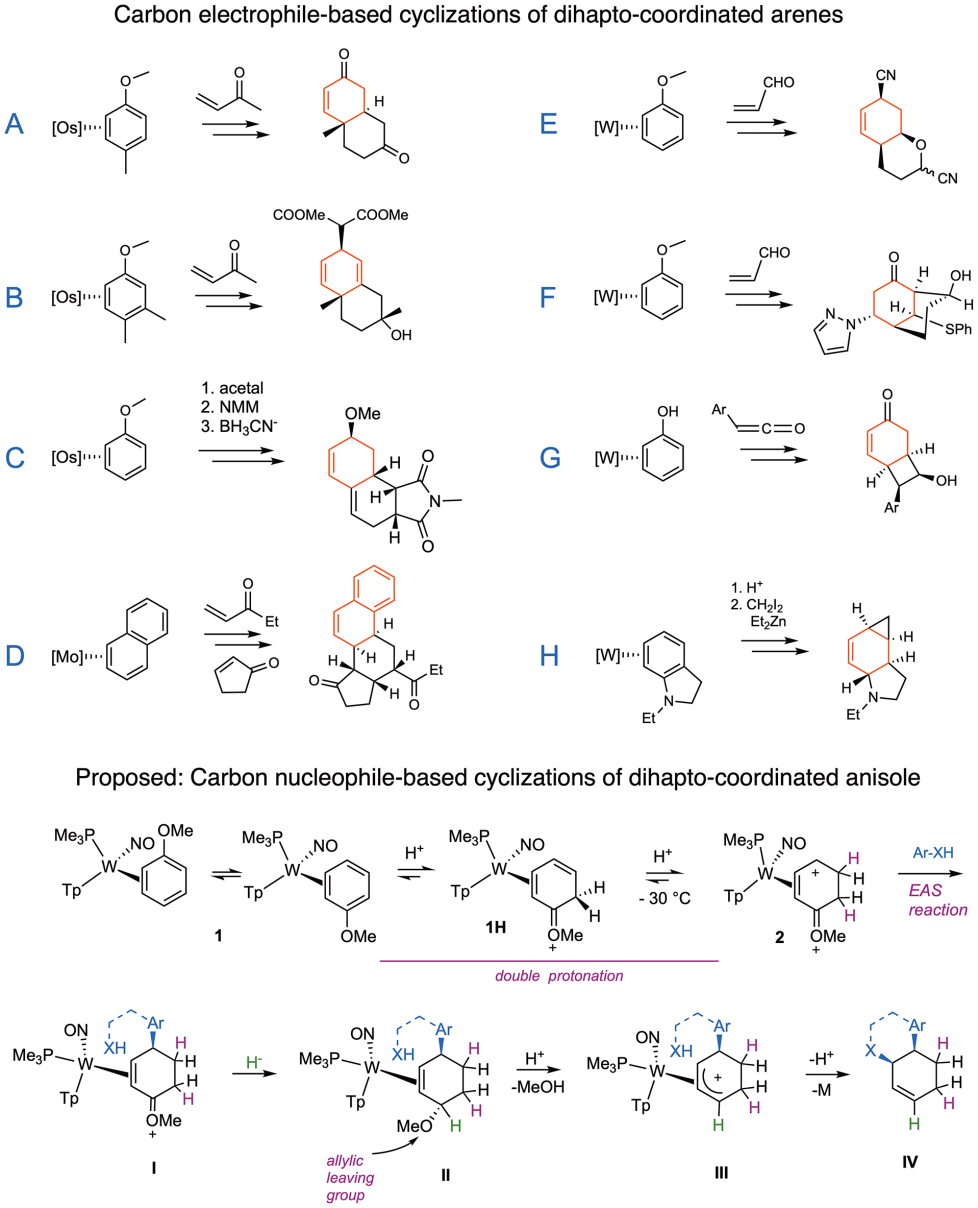
Electrophile-based cyclizations of η^2^-arene complexes (A–H) and the proposed π-nucleophile-based cyclization of an η^2^-anisole complex (**I** → **IV**). NMM = *N*-methylmaleimide.

## Results and Discussion

For this study, we chose a range of commercially available aromatic compounds to generate complexes **3–9**, all of which contain a potential pendant nucleophile (Figure 2, red) that could be used in a subsequent cyclization (**IV** in Figure 1). In a typical procedure, the anisole complex **1** was dissolved in CH_3_CN then combined with triflic acid (HOTf) at −30 °C to generate the 2*H*-anisolium complex **1H** (Figure 1) as a single diastereomer. Subsequent exposure of **1H** to HOTf provides the dicationic intermediate **2**. A solution of the arene (CH_3_CN; −30 °C) was then added. Several *p*-substituted phenols were chosen, which were found to undergo selective addition to the dicationic intermediate **2** at the ortho carbon of the phenol to form the oxocarbenium triflate salts (**3–7**; Figure 2).^17^ Treatment of **3–7** with a methanolic solution of sodium borohydride (NaBH_4_) followed by acidolysis with HOTf generated the π-allyl species **10–13** (Figure 3).^19^ Subsequent treatment of **10–13** with base effected the ring-closure to generate tricyclic ethers **14–17** (Figure 3). These tetrahydrodibenzofuran products were all formed as single regio- and stereoisomers. Their assigned structures (Figure 3) were supported by COSY, NOESY, HSQC, and HMBC data (Supporting Information), which indicate that addition of both the arene ring and the oxygen occur anti to metal coordination, and proximal to the PMe_3_ ligand. Single crystal X-ray diffraction (SC-XRD) studies on **14**, **15**, and **17** confirm the predicted structures for these compounds (Figure 4 and Supporting Information).

**Figure 2:**
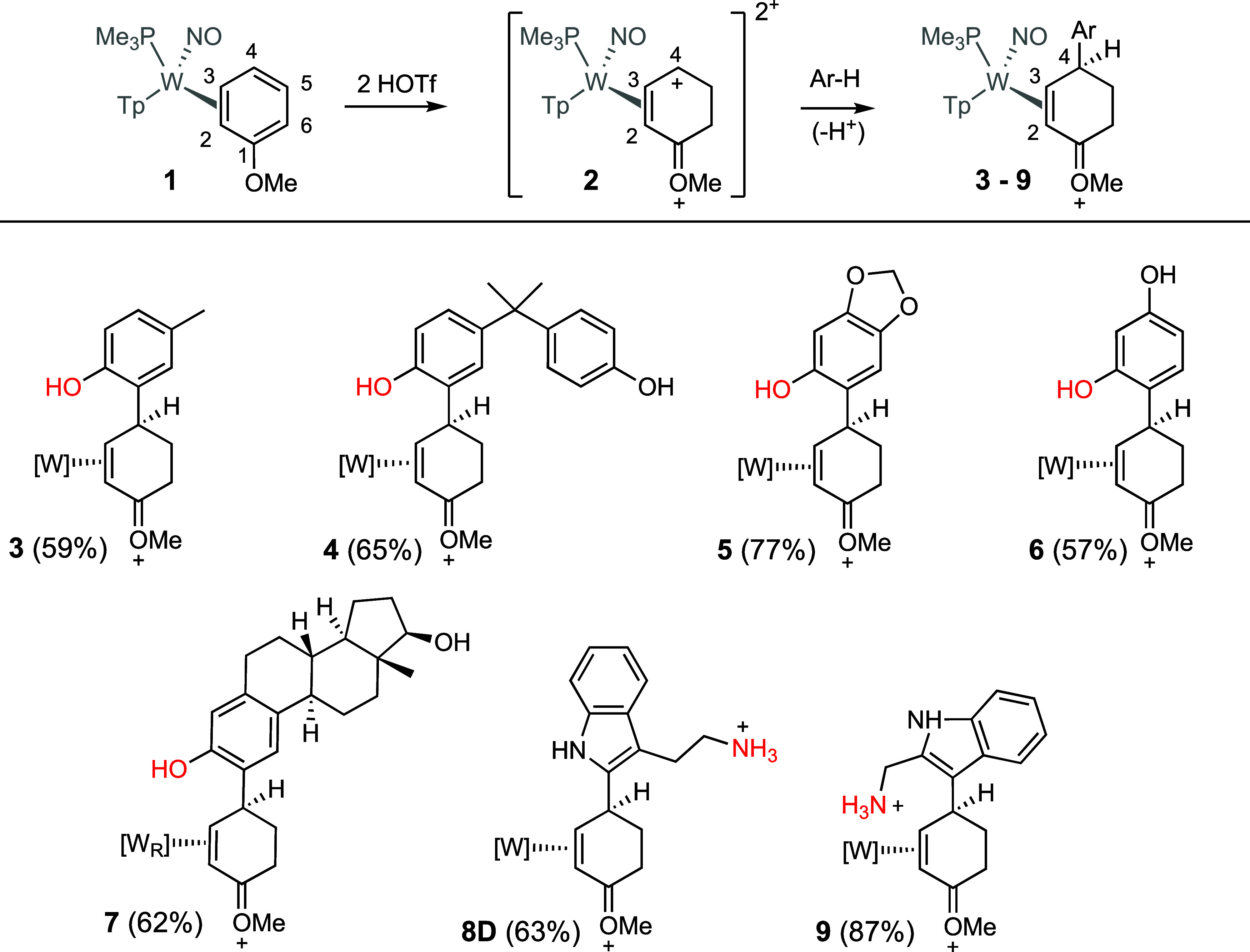
Phenol and indole addition products resulting from their addition to the double-protonated anisole complex (**2**). For all isolated salts **3–9**, the counterion is OTf^–^.

**Figure 3:**
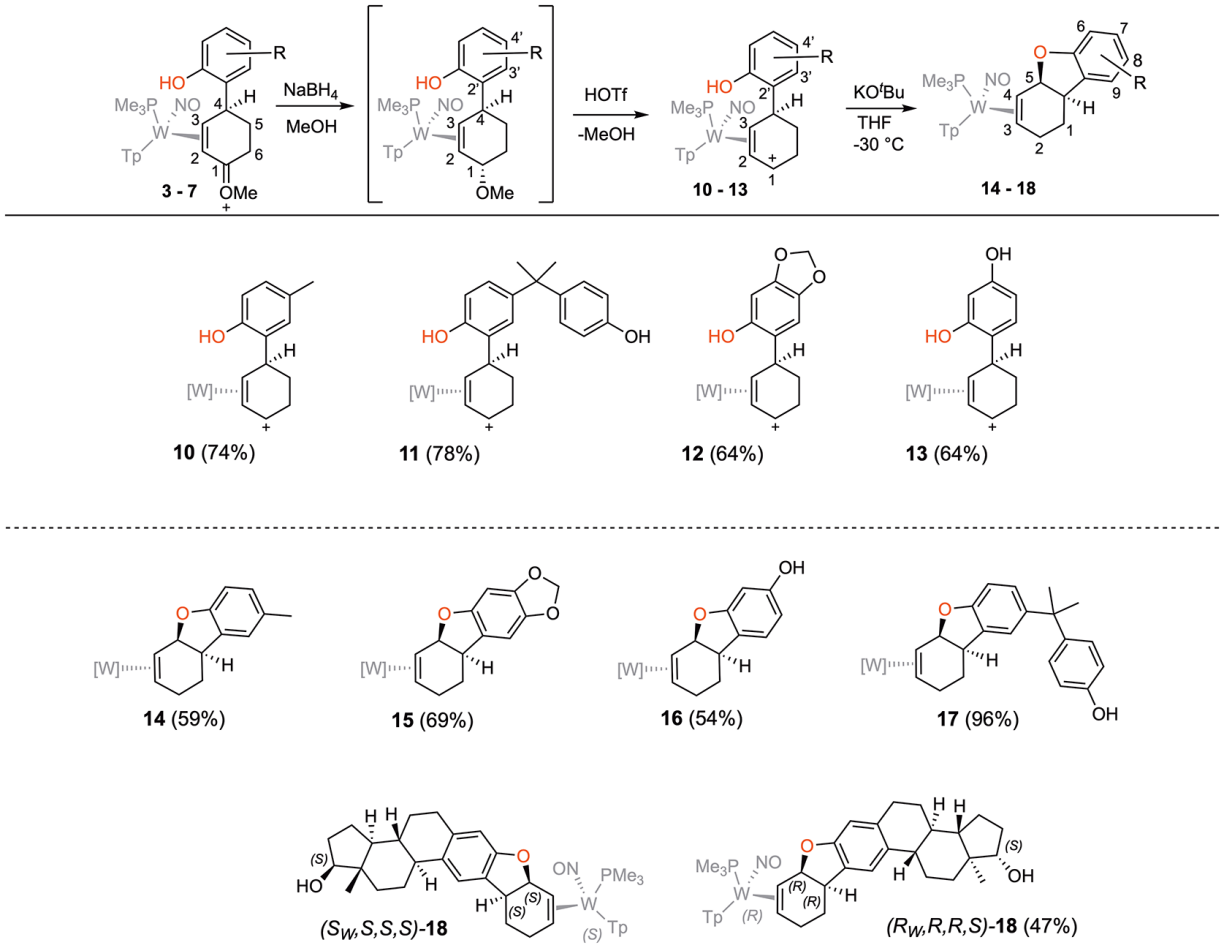
Cyclizations of the phenol derivatives to tetrahydrodibenzofuran complexes. [W] = WTp­(NO)­(PMe_3_). For the salts **10–13**, the counterion is OTf^–^.

**Figure 4:**
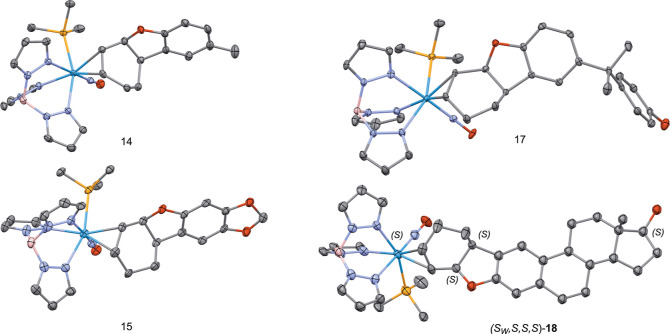
Molecular structures of polycyclic dihydrobenzofuran complexes **14**, **15**, and **17** and (*S*_*W*_,*S*,*S*,*S*)-**18**. 30% ellipsoids. Hydrogen atoms have been removed for clarity, as has cocrystallized solvent in **15** and **17** and the minor position of the disorder in **18**.

When β-estradiol is used as the phenolic reagent, two diastereomers were formed in a 1:1 ratio, owing to the racemic mixture of enantiomers that make up the anisole complex **1** (and correspondingly, **2**). The analogous reduction, acidolysis and ring-closure resulted in two diastereomers of the hexacyclic compound **18**. A SC-XRD study of a single crystal grown from **18** revealed that these two diastereomers cocrystalized, and the structure of (*S*_*W*_,*S*,*S*,*S*)-**18** is shown in Figure 4. Repeating this procedure with a sample of **1** enriched to have an R: S ratio of 9:1 generated (*R*_*W*_,*R*,*R*,*S*)-**18** in the same 9:1 ratio.

The tetrahydrodibenzofuran moiety is a common feature in natural products and biologically active compounds.^20–22^ These moieties have be prepared by several methods including cycloaddition of phenols to alkenes,^23^ palladium-catalyzed annulation of 1,3-dienes,^21,24^ and various photochemical approaches.^25^ In many cases, organic alkenes can be liberated from [WTp­(NO)­(PMe_3_)] (where Tp = hydridotris­(pyrazolyl)­borate), denoted here on as [W], using chemical oxidants such as Ag^+^, DDQ, and NOPF_6_. However, the tetrahydrodibenzofuran products **14–18** present a challenge in that a phenolic oxygen is a good leaving group which occupies the allylic position of the alkene complex. Consequently, our attempts to decomplex the tetrahydrodibenzofurans were thwarted by ring-opening to reform the corresponding π-allyl complexes (**10–13**).

We also explored other heterocyclic ring closures using this methodology. Indoles substituted at the 2-position served as a convenient scaffold for this endeavor. For example, derivative **9** has a pendant methanamine which could serve as the ring-closing nucleophile (Figure 5). After reduction of **9** to **19**, and removal of methanol through acidolysis to form **20**, treatment of **20** with LiHMDS at −60 °C afforded only the cyclization product **21**. Attempts to oxidatively decomplex the organic heteropolycyclic were again challenging, however, utilizing a Br_2_ solution (1.0 M, 1,2-dichloroethane), oxidative decomplexation of **22** was accomplished in an isolated yield of 17%.

**Figure 5:**
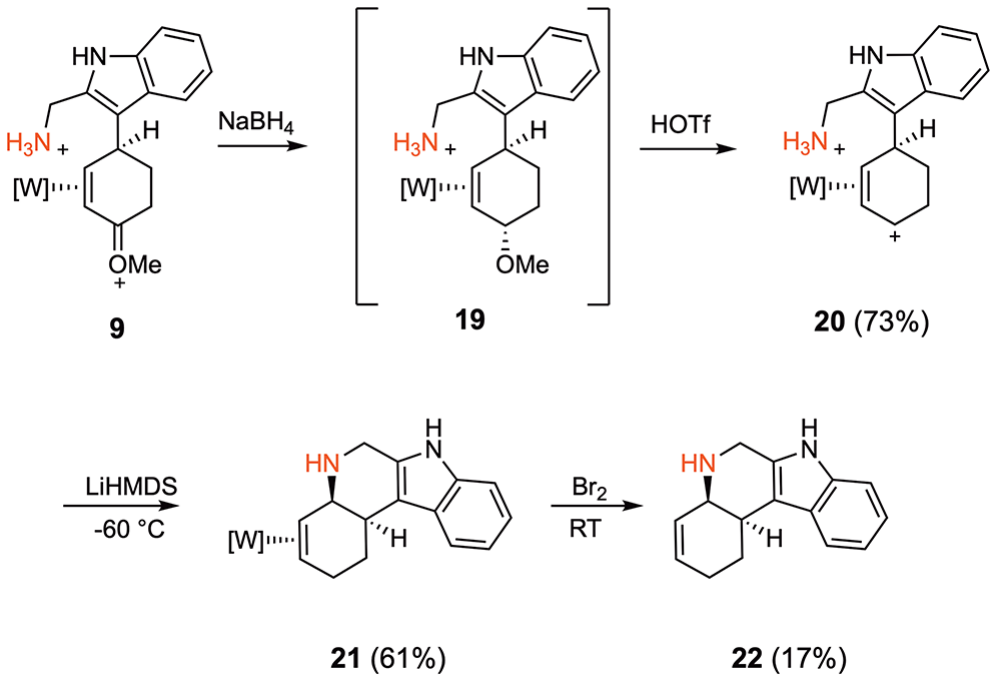
Cyclization of 2-indole-derived complex **20**. The counterion is OTf^–^.

Generation of an additional alkaloid core was also attempted using the tryptamine derivative **8D**. Reduction followed by acidolysis formed the allyl complex **24D** (Figure 6). When this compound was treated with base (LiHDMS; −60 °C), the intended cyclization occurred (**26D**), but the major product recovered was the diene complex **25D** (1.5:1; **25D**:**26D**; Figure 6). Using other bases or temperatures failed to improve the situation. A previous DFT analysis from our group of a simplified cyclohexenyl complex provided some guidance in our synthetic design.^19^ π-Allyl complexes of {WTp­(NO)­(PMe_3_)} exist as two interconverting conformers, of an “η^2^-allyl species” differing by W-C bond distances within the allyl moiety (Figure 6).^19^ Formation of tetracyclic **26D** necessitates that **24D** undergoes an allyl shift from a distal η^2^-allyl complex to a proximal η^2^-allyl complex (**24P**). For the parent cyclohexenyl complex (C_6_H_9_^+^), a proximal allyl complex is ∼4 kcal/mol higher in energy than its distal conformer. This energy difference is likely reflected in the transition state for the desired addition reaction. Thus, we speculated that accessing the alternative coordination diastereomer of the tryptamine oxocarbenium (**8P**),^18^ (with the oxocarbenium carbon proximal to the PMe_3_) would ultimately favor the distal conformer of the allyl complex, **24P**, which would be properly aligned for the desired ring-closure. This species can be obtained by taking advantage of the observation that in its crystalline form, **1** exists exclusively as the proximal isomer. Trapping **1** at low temperature (−30 °C) through double protonation (**2P** in Figure 6) followed by addition of tryptamine results in formation of the proximal isomer **8P**.^17^

**Figure 6:**
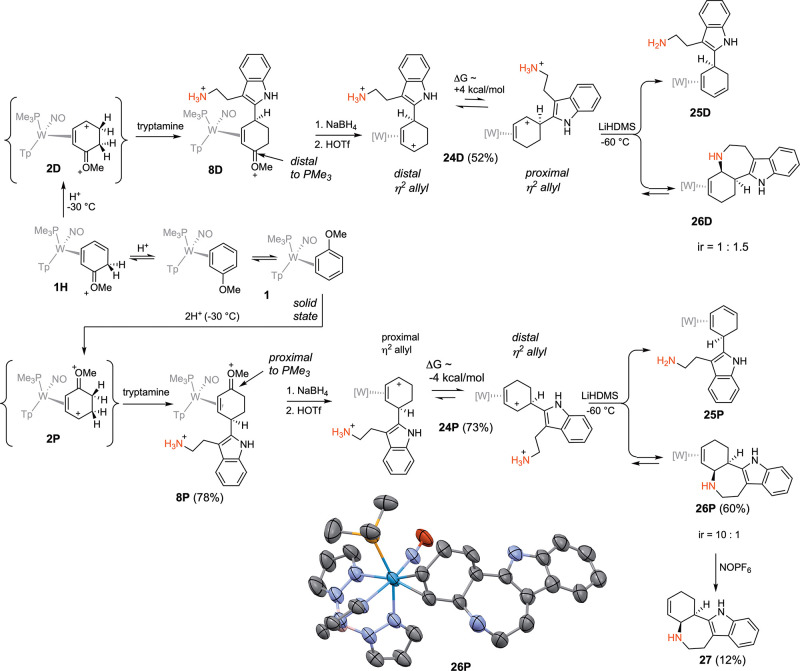
Cyclization of tryptamine-derived complexes. For the SC-XRD structure of **26P**: 30% ellipsoids. Hydrogen atoms have been removed for clarity. The counterion is OTf^–^.

Gratifyingly, using LiHDMS as the base, a ∼10:1 isomer ratio (ir) of **26P**:**25P** was obtained, where the major product is now the desired tetracyclic (**26P**). A crystal structure obtained for **26P** confirms its expected structure (Figure 6). The tetracyclic alkaloid **27** shares the same tetracyclic core as many of the ibogaine alkaloids,^26^ including the fused-ring tetracyclic Ervaoffine K,^27^ and does not appear to have been made from a benzene derivative previously. However, the liberation of the organic **27** from the complex **26P** was again achieved in only modest yield (12%; NOPF_6_).

There is a rich chemistry of η^6^-arene complexes undergoing nucleophilic additions including complexes of Cr, Mo, Mn, Re, Ru, Rh, and Ir.^28^ Of these, Cr­(CO)_3_ ^29^ and [Mn­(CO)_3_]^+^ ^30^ have the most extensive history of application to organic synthesis. Recent examples of hydrofunctionalization of η^6^-benzenes can be found in elegant work by the Li group,^31–33^ which notably includes the synthesis of tetrahydrodibenzofurans.^33^ Remarkably, only a handful of examples exist in which two groups have been added to the arene complex thereby forming a bicyclic species. These include Semmelhack’s synthesis of acorenone,^34^ Rudler’s lactonization of 2-phenylpyridine,^35^ Cooper’s nitrone cyclization,^36^ and Wulff’s benzannulation/nucleophilic addition sequence.^37^ Heteroannulation promoted by the η^2^-coordination of arenes has also been reported previously, enabling the synthesis of lactams and chromane cores.^38–40^ The results presented herein extend on the use of [WTp­(NO)­(PMe_3_)] as a means to access polycyclic compounds through the utilization of EAS reactions. This study demonstrates the feasibility of an intramolecular variation of the functionalization of an η^2^-anisole complex via its double-protonation, and points to a potentially useful synthetic approach to organic heteropolycyclics provided that an alternative organometallic promoter is developed that allows for a more facile decomplexation. The related system MoTp­(NO)­(DMAP)­(η^2^-benzene) has been recently reported to undergo double-protonation and subsequent EAS with anisole, generating a species similar to **III** in Figure 1 in which ArH is anisole and {WTp­(NO)­(PMe_3_)} is replaced with {MoTp­(NO)­(DMAP)}.^18^ Perhaps this system could be adapted to effect ring-closures similar to those described herein.

### Experimental Procedures and Characterizations

#### General Methods

NMR spectra were obtained on 400, 600 or 800 MHz spectrometers. Chemical shifts are referenced to tetramethylsilane (TMS) utilizing residual ^1^H or ^13^C signals of the deuterated solvents as internal standards. Chemical shifts are reported in ppm and coupling constants (*J*) are reported in hertz (Hz). Electrochemical experiments were performed under a nitrogen atmosphere. All synthetic reactions were performed in a glovebox under a dry nitrogen atmosphere unless otherwise noted. All solvents were sparged with nitrogen prior to use. Deuterated solvents were used as received from Cambridge Isotopes. When possible, pyrazole (Pz) protons of the hydrido­(trispyrazolyl)­borate (Tp) ligand were assigned as “Pz3/5 or Pz4”. B-H peaks (around 4–5 ppm) in the ^1^H NMR spectra are not assigned due to their quadrupole broadening. Compounds **1**, **2**, **3**, **4**, **5**, **6**, **7D/P**, **8** and **24D/P** were previously reported.^16^

#### General Procedure **1**

To a test tube was added **X** and MeOH (5 mL). This solution was chilled to −30 °C for 5 min before adding NaBH_4_. The reaction mixture was allowed to stir at −30 °C for 30 min before diluting with DCM (5 mL) and washing with DI H2O (2 × 5 mL). The organic layer was dried over anhydrous Na_2_SO_4_ and concentrated in vacuo. A solution of HOTf in DME was added to the resulting oil to give a dark red homogeneous solution. This solution was then added to stirring Et_2_O (300 mL) to produce a precipitate which was collected on a medium-porosity fritted funnel, washed with Et_2_O (30 mL) and desiccated under static vacuum overnight.

#### General Procedure **2**

To a test tube was added **X** and THF. This solution was chilled to −30 °C for 5 min before adding a solution of K­(t-BuO) (20% w/w in THF) or TEA. The reaction mixture was allowed to stir at room temperature for 30 min before diluting with DCM (5 mL) and washing with saturated Na_2_CO_3_ (2 × 5 mL). The organic layer was dried over anhydrous Na_2_SO_4_ and concentrated in vacuo. Film dissolved in minimal DCM and added to 150 mL of stirring pentanes to produce a precipitate which was collected on a medium-porosity fritted funnel and desiccated under static vacuum overnight.
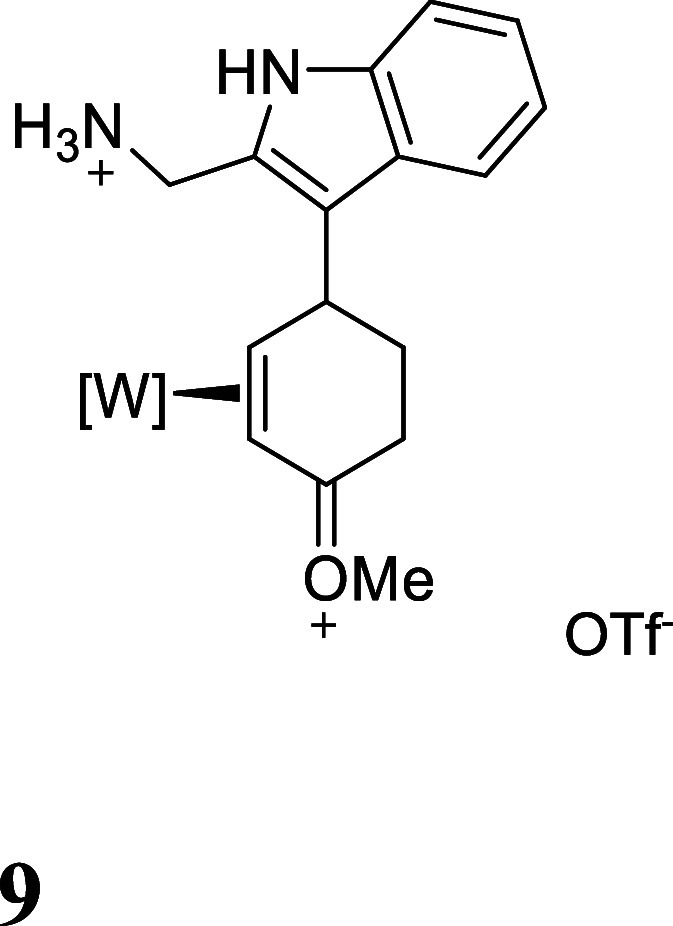


To a 34 mL test tube were added **1** and MeCN (5 mL). This solution was chilled to −30 °C for 5 min before adding a chilled (−30 °C) solution of HOTf in MeCN (0.118 g, 0.788 mmol). To a chilled (−30 °C) solution of 1*H*-indole-2-methanamine (0.230 g, 1.58 mmol) in MeCN (3 mL) was added a chilled (−30 °C) solution of HOTf in MeCN (0.118 g, 0.788 mmol). This solution was then added to the solution containing **1**. The reaction mixture was then diluted with DCM (5 mL) and washed with DI H_2_O (3 × 5 mL). The organic layer was dried over anhydrous MgSO_4_ and concentrated in vacuo. The film was dissolved in minimal DCM and added to stirring Et_2_O (150 mL). The resulting precipitate was collected on a fine porosity fritted funnel and washed with Et_2_O (30 mL). Tan solid (0.313 g (87.7%))

^1^H NMR (400 MHz, CD_2_Cl_2_) δ: 10.06 (s, 1H), 8.15 (d, *J* = 2.1 Hz, 1H), 7.88 (d, *J* = 2.1 Hz, 1H), 7.87–7.82 (m, 4H), 7.67 (d, *J* = 8.0 Hz, 1H), 7.45 (d, *J* = 7.8 Hz, 2H), 7.23 (t, *J* = 7.4 Hz, 1H), 7.15 (t, *J* = 7.3 Hz, 1H), 6.48 (t, *J* = 2.2 Hz, 1H), 6.44 (t, *J* = 2.3 Hz, 1H), 6.35 (t, *J* = 2.3 Hz, 1H), 5.01–4.89 (m, 1H), 4.85 (d, *J* = 14.5 Hz, 1H), 4.67 (d, *J* = 14.5 Hz, 1H), 4.22 (dd, *J* = 15.8, 8.2 Hz, 1H), 3.54 (d, *J* = 7.8 Hz, 1H), 3.31–3.19 (m, 1H), 3.08 (s, 3H), 2.82 (d, *J* = 17.7 Hz, 1H), 2.28–2.19 (m, 1H), 1.94–1.79 (m, 1H), 0.98 (d, *J* = 9.3 Hz, 9H). ^13^C NMR (101 MHz, CD_2_Cl_2_) δ: 194.3, 145.0, 144.2, 142.5, 139.1, 138.8, 138.5, 137.0, 126.7, 124.9, 123.7, 122.3, 120.3, 118.6, 112.6, 108.7, 108.4, 107.9, 75.3 (d, *J* = 14.4 Hz), 66.9, 57.9, 37.1, 35.0, 33.99, 30.2, 14.1 (d, *J* = 31.4 Hz). ESI-HRMS (*m*/*z*): [M]^+^ calculated for C_28_H_38_BN_9_O_2_PW^+^, 758.2483; found, 758.2478.
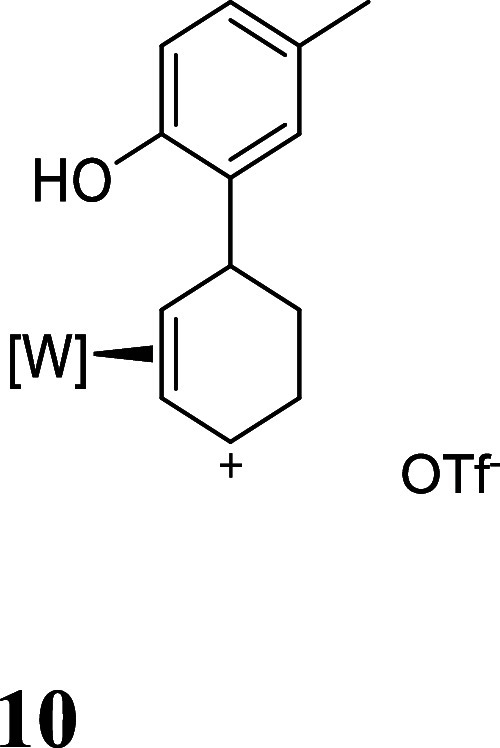


Used general procedure **1** with **3** (0.3112 g, 0.3580 mmol), NaBH_4_ (0.2242 g, 5.927 mmol), and HOTf (0.1862 g, 1.241 mmol) in DME (1 mL). Tan solid (0.2227 g, (74%)).

^1^H NMR (800 MHz, CD_3_CN) δ: 8.42 (d, *J* = 2.3 Hz, 1H), 8.17 (d, *J* = 2.3 Hz, 1H), 8.00 (d, *J* = 2.3 Hz, 1H), 7.95 (d, *J* = 2.4 Hz, 1H), 7.94 (d, *J* = 2.3 Hz, 1H), 7.82 (d, *J* = 2.5 Hz, 1H), 7.28 (br s, 1H), 6.96 (br s, 1H), 6.93 (d, *J* = 8.2, 1H), 6.74 (d, *J* = 7.9 Hz, 1H), 6.52 (m, 3H)*, 6.35 (t, *J* = 2.5 Hz, 1H), 5.37 (t, *J* = 7.5 Hz, 1H), 4.35 (dd, *J* = 15.9, 7.2 Hz, 1H), 4.28 (dd, *J* = 11.3, 5.9 Hz, 1H), 3.51 (ddd, *J* = 18.7, 10.9, 6.2 Hz, 1H), 3.32 (dt, *J* = 19.7, 6.4 Hz, 1H), 2.31 (s, 3H), 1.72 (dt, *J* = 12.5, 6.0 Hz, 1H), 1.21–1.13 (m, 1H), 1.01 (d, *J* = 9.9 Hz, 9H). ^13^C NMR (201 MHz, CD_3_CN) δ: 151.9, 149.4, 146.2, 143.4, 139.7, 139.6, 139.5, 135.2, 133.9, 131.1, 129.7, 129.1, 116.1, 109.6, 109.0, 108.2, 105.7 (d, *J*_CP_ = 3.3 Hz), 76.2 (d, *J*_CP_ = 12.1 Hz), 36.7, 30.3, 26.3, 20.8, 13.7 (d, *J*_CP_ = 32.6 Hz). APCI-HRMS (*m*/*z*): [M]^+^ calculated for [C_25_H_34_BN_7_O_2_PW]^+^, 690.2108; found, 690.2129. Composition confirmed by single-crystal X-ray diffraction.
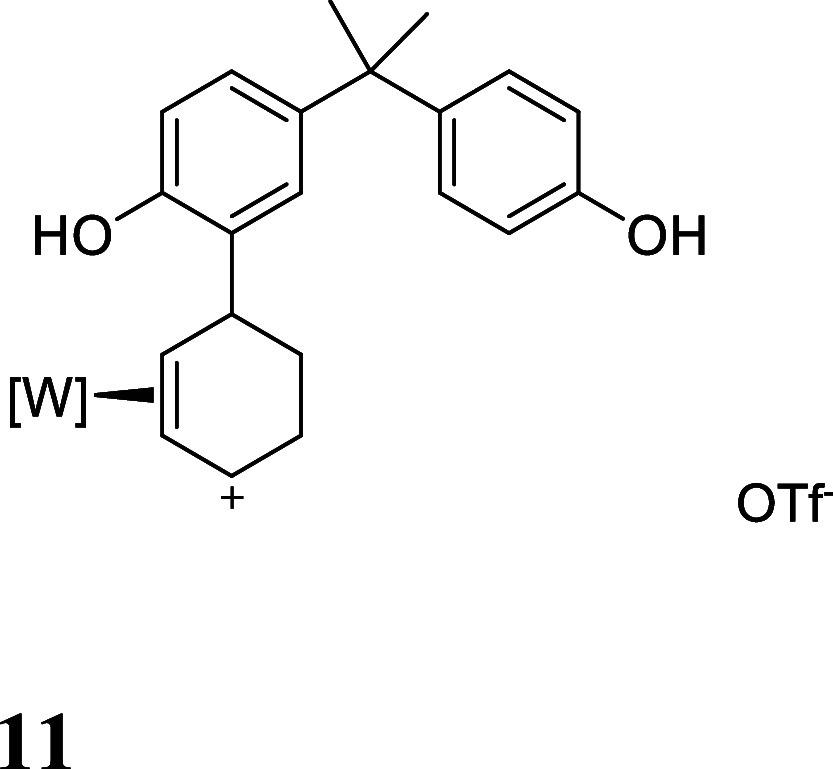


Used general procedure **1** with **4** (1.3440 g, 1.3583 mmol), NaBH_4_ (0.5034 g, 13.31 mmol), and HOTf (0.8603 g, 5.732 mmol) in DME (3 mL). Tan solid (1.0100 g, (78%)).

^1^NMR (800 MHz, acetone) δ: 8.59 (d, *J* = 2.3 Hz, 1H), 8.35 (d, *J* = 2.3 Hz, 1H), 8.19 (d, 1H), 8.14 (d, *J* = 0.8 Hz, 1H), 7.97 (d, *J* = 0.8 Hz, 1H), 7.88 (d, *J* = 2.3 Hz, 1H), 7.21 (s, 1H), 7.10 (d, *J* = 8.7 Hz, 1H), 7.07 (dd, *J* = 8.4, 2.4 Hz, 1H), 6.85 (d, *J* = 8.4 Hz, 1H), 6.79 (s, 1H), 6.74 (td, *J* = 7.5, 2.1 Hz, 1H), 6.67 (t, *J* = 2.3 Hz, 1H), 6.63 (t, *J* = 2.3 Hz, 1H), 6.41 (t, *J* = 2.4 Hz, 1H), 5.43 (t, *J* = 1.4 Hz, 1H), 4.34 (dd, *J* = 16.0, 7.2 Hz, 1H), 4.30 (dd, *J* = 10.9, 5.6 Hz, 1H), 3.62–3.50 (m, 1H), 2.91 (s, 1H), 1.79 (dt, *J* = 12.4, 6.0 Hz, 1H), 1.65 (s, 6H), 1.32–1.29 (m, 2H), 1.11 (d, *J* = 9.9 Hz, 9H). ^13^C NMR (201 MHz, Acetone) δ: 156.0, 152.5, 149.4, 146.4, 143.2, 142.9, 139.5, 139.4, 134.3, 128.6, 128.5, 128.4, 126.0, 115.6, 115.5, 109.6, 109.3, 108.1, 105.0, 75.8 (d, *J* = 12.4 Hz), 42.4, 38.2, 31.4, 31.2, 26.2, 13.5 (d, *J* = 32.1 Hz). ESI-HRMS (*m*/*z*): [M]^+^ calculated for C_33_H_42_BN_7_O_3_PW^+^, 810.2684; found, 810.2671.
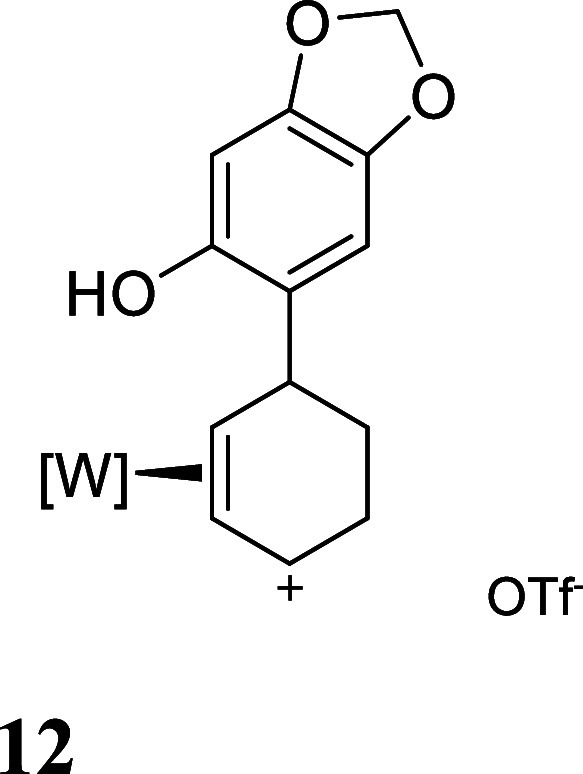


Used general procedure **1** with **5** (0.2408 g, 0.2678 mmol), NaBH_4_ (0.1282 g, 3.389 mmol), and HOTf (0.1109 g, 0.7389 mmol) in DME (1 mL). Tan solid (0.1489 g, (64%)).

^1^H NMR (800 MHz, DMSO) δ: 9.30 (s, 1H), 8.55 (d, *J* = 2.3 Hz, 1H), 8.34 (d, *J* = 2.4 Hz, 1H), 8.33 (d, *J* = 2.3 Hz, 1H), 8.25 (d, *J* = 2.3 Hz, 1H), 8.22 (d, *J* = 2.4 Hz, 1H), 8.04 (d, *J* = 2.5 Hz, 1H), 7.01 (s, 1H), 6.60 (t, *J* = 2.3 Hz, 1H), 6.58 (t, *J* = 2.3 Hz, 1H), 6.49 (t, *J* = 7.0 Hz, 1H), 6.45 (s, 1H), 6.44 (t, *J* = 2.3 Hz, 1H), 5.96 (d, *J* = 1.1 Hz, 1H), 5.91 (d, *J* = 1.1 Hz, 1H), 5.37 (t, *J* = 7.3 Hz, 1H), 4.45 (dd, *J* = 16.1, 7.3 Hz, 1H), 4.24 (dd, *J* = 11.3, 6.0 Hz, 1H), 3.48–3.40 (m, 1H), 3.31–3.25 (m, 1H), 1.63–1.59 (m, 1H), 1.03 (d, *J* = 10.0 Hz, 9H), 0.99 (d, *J* = 8.4 Hz, 1H). ^13^C NMR (201 MHz, DMSO) δ: 148.6, 147.9, 145.6, 141.5, 141.0, 140.3, 138.7, 138.5, 131.3, 126.8, 126.0, 108.7, 108.1, 107.3, 107.3, 104.9, 100.6, 97.2, 75.3 (d, *J* = 12.0 Hz), 35.0, 29.4, 25.1, 12.6 (d, *J* = 31.8 Hz). ESI-HRMS (*m*/*z*): [M]^+^ calculated for C_25_H_32_BN_7_O_4_PW^+^, 720.1850; found, 720.1856.
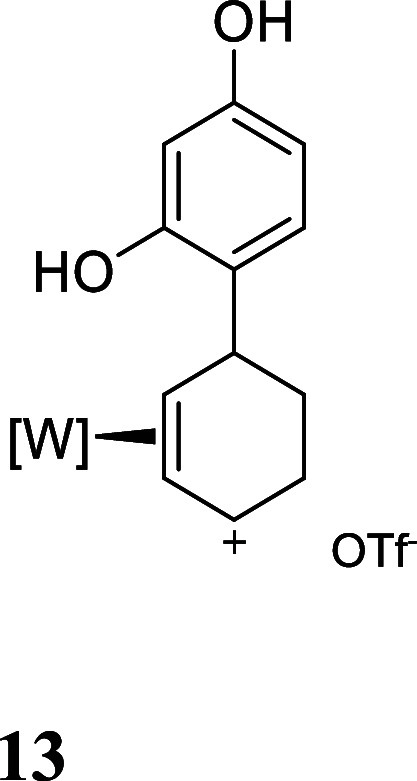


Used general procedure **1** with **6** (0.140 g, 0.160 mmol), NaBH_4_ (0.0485 g, 1.28 mmol), and HOTf (0.0722 g, 0.481 mmol) in DME (3 mL). Tan solid (0.0859 g, (64%)).

^1^H NMR (400 MHz, CD_2_Cl_2_) δ: 8.41 (d, *J* = 2.3 Hz, 1H), 8.08 (d, *J* = 2.3 Hz, 1H), 7.94 (d, *J* = 2.3 Hz, 1H), 7.88 (d, *J* = 0.8 Hz, 1H), 7.80 (d, *J* = 2.3 Hz, 1H), 7.75 (dt, *J* = 2.5, 0.8 Hz, 1H), 7.17 (d, *J* = 8.3 Hz, 1H), 6.60 (d, *J* = 2.4 Hz, 1H), 6.58–6.55 (m, 1H), 6.55–6.53 (m, 2H), 6.45 (t, *J* = 6.8 Hz, 1H), 6.36 (t, *J* = 2.4 Hz, 1H), 5.30 (t, *J* = 7.4 Hz, 1H), 4.36 (dd, *J* = 15.8, 7.2 Hz, 1H), 4.23 (dd, *J* = 10.9, 6.0 Hz, 1H), 3.64–3.53 (m, 1H), 3.42–3.31 (m, 1H), 1.84–1.72 (m, 1H), 1.45–1.27 (m, 1H), 1.11 (d, *J* = 9.7 Hz, 9H). ^13^C NMR (101 MHz, CD_2_Cl_2_) δ: 156.2, 154.2, 147.8, 144.9, 141.5, 138.3, 138.1, 131.5, 128.6, 125.7, 108.5, 108.3, 108.2, 107.1, 104.5 (d, *J* = 3.5 Hz), 103.4, 76.4 (d, *J* = 12.0 Hz), 36.2, 28.7, 25.5, 13.4 (d, *J* = 32.3 Hz). ESI-HRMS (*m*/*z*): [M]^+^ calculated for C_24_H_32_BN_7_O_3_PW, 692.1901; found, 692.1923.
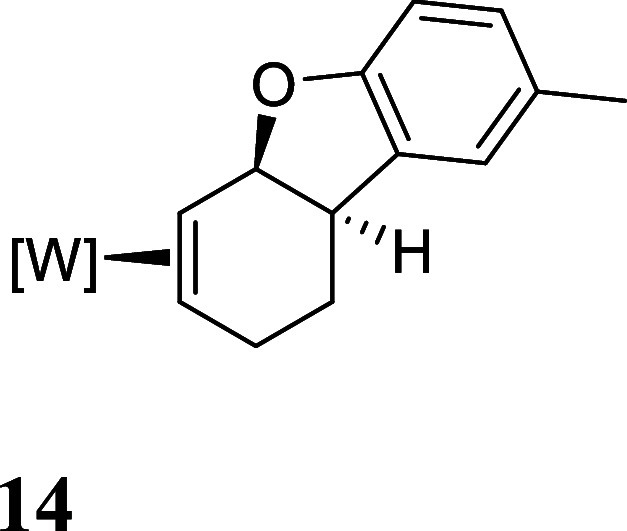


Used general procedure **2** with **10** (0.2227 g, 0.2653 mmol) and K­(t-BuO) (20% w/w in THF) (0.5 mL, 0.8 mmol). Tan solid (0.1075 g, (59.0%)).

^1^H NMR (600 MHz, CD_3_CN) δ: 8.16 (d, *J* = 2.1 Hz, 1H), 8.06 (d, *J* = 2.0 Hz, 1H), 7.89 (d, *J* = 2.4 Hz, 1H), 7.82 (d, *J* = 2.4 Hz, 1H), 7.77 (d, *J* = 2.4 Hz, 1H), 7.47 (d, *J* = 2.2 Hz, 1H), 7.06 (s, 1H), 6.91 (d, *J* = 8.1 Hz, 1H), 6.64 (d, *J* = 8.0 Hz, 1H), 6.40 (t, *J* = 2.2 Hz, 1H), 6.28 (t, *J* = 2.2 Hz, 1H), 6.25 (t, *J* = 2.2 Hz, 1H), 5.52 (d, *J* = 7.1 Hz, 1H), 3.23–3.18 (m, 1H), 2.91–2.81 (m, 2H)*, 2.72–2.66 (m, 1H), 2.26 (s, 3H), 2.01–1.98 (m, 1H), 1.37–1.25 (m, 2H)*, 1.14 (d, *J* = 8.4 Hz, 9H). ^13^C NMR (151 MHz, CD_3_CN) δ: 158.6, 144.2, 143.5, 141.8, 137.9, 137.4, 137.2, 135.8, 130.0, 128.9, 126.2, 109.4, 107.7, 107.2, 106.8, 89.9 (d, *J*_CP_ = 5.1 Hz), 53.8 (d, *J*_CP_ = 10.3 Hz), 52.5, 42.7, 30.5, 27.9, 21.0, 12.8 (d, *J*_CP_ = 28.0 Hz). Composition confirmed by single-crystal X-ray diffraction.
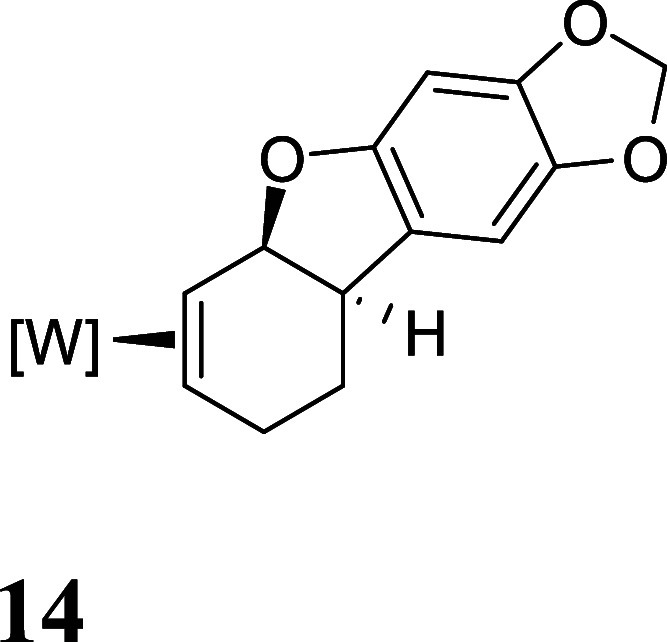


Used general procedure **2** with **13** (0.1419 g, 0.1632 mmol) and K­(t-BuO) (20% w/w in THF) (0.25 mL, 0.4 mmol). Tan solid (0.081 g, (69%)).

^1^H NMR (800 MHz, DMSO) δ: 8.08 (d, *J* = 2.0 Hz, 1H), 8.07 (d, *J* = 2.4 Hz, 1H), 8.05 (d, *J* = 1.9 Hz, 1H), 8.00 (d, *J* = 2.3 Hz, 1H), 7.92 (d, *J* = 2.4 Hz, 1H), 7.56 (d, *J* = 2.1 Hz, 1H), 6.85 (s, 1H), 6.48 (s, 1H), 6.46 (t, *J* = 0.9 Hz, 1H), 6.34 (t, *J* = 0.8 Hz, 1H), 6.29 (t, *J* = 0.9 Hz, 1H), 5.90 (d, *J* = 1.0 Hz, 3H), 5.45 (d, *J* = 7.1 Hz, 1H), 3.10–3.05 (m, 1H), 2.83–2.74 (m, 1H), 2.64–2.58 (m, 1H), 1.90–1.84 (m, 1H), 1.25–1.22 (m, 3H), 1.12–1.09 (m, 12H). ^13^C NMR (201 MHz, DMSO) δ: 146.2, 142.8, 141.7, 140.5, 140.2, 136.7, 136.2, 136.0, 125.1, 106.6, 106.2, 105.6, 104.8, 100.3, 92.4, 89.2 (d, *J* = 5.2 Hz), 52.0 (d, *J* = 10.1 Hz), 50.9, 41.1, 29.3, 26.4, 11.6 (d, *J* = 27.8 Hz). ESI-HRMS (*m*/*z*): [M]^+^ calculated for C_25_H_31_BN_7_O_4_PW, 719.1778; found C_25_H_32_BN_7_O_4_PW^+^, 720.1848, corresponding to reformation of **13**.
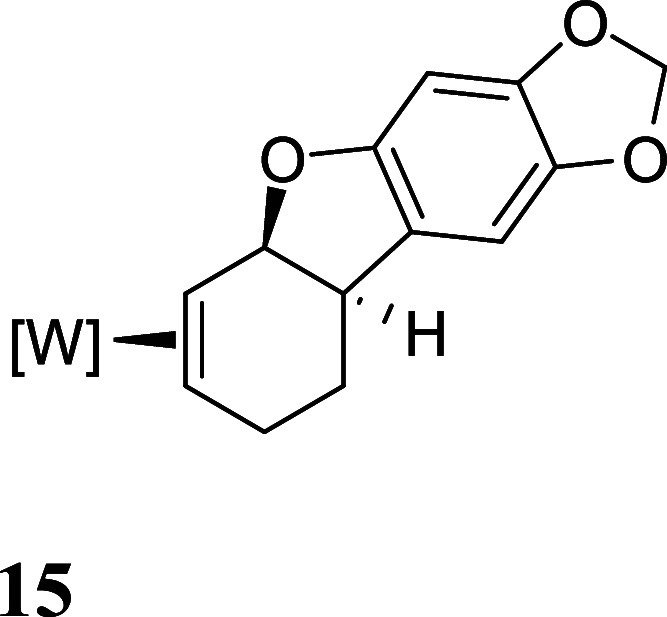


Used general procedure **2** with **12** (0.1419 g, 0.1632 mmol) and K­(t-BuO) (20% w/w in THF) (0.25 mL, 0.4 mmol). Tan solid (0.081 g, (69%)).

^1^H NMR (800 MHz, DMSO) δ: 8.08 (d, *J* = 2.0 Hz, 1H), 8.07 (d, *J* = 2.4 Hz, 1H), 8.05 (d, *J* = 1.9 Hz, 1H), 8.00 (d, *J* = 2.3 Hz, 1H), 7.92 (d, *J* = 2.4 Hz, 1H), 7.56 (d, *J* = 2.1 Hz, 1H), 6.85 (s, 1H), 6.48 (s, 1H), 6.46 (t, *J* = 0.9 Hz, 1H), 6.34 (t, *J* = 0.8 Hz, 1H), 6.29 (t, *J* = 0.9 Hz, 1H), 5.90 (d, *J* = 1.0 Hz, 3H), 5.45 (d, *J* = 7.1 Hz, 1H), 3.10–3.05 (m, 1H), 2.83–2.74 (m, 1H), 2.64–2.58 (m, 1H), 1.90–1.84 (m, 1H), 1.25–1.22 (m, 3H), 1.12–1.09 (m, 12H). ^13^C NMR (201 MHz, DMSO) δ: 146.2, 142.8, 141.7, 140.5, 140.2, 136.7, 136.2, 136.0, 125.1, 106.6, 106.2, 105.6, 104.8, 100.3, 92.4, 89.2 (d, *J* = 5.2 Hz), 52.0 (d, *J* = 10.1 Hz), 50.9, 41.1, 29.3, 26.4, 11.6 (d, *J* = 27.8 Hz). ESI-HRMS (*m*/*z*): [M]^+^ calculated for C_25_H_31_BN_7_O_4_PW, 719.1778; found C_25_H_32_BN_7_O_4_PW^+^, 720.1848, corresponding to reformation of **13**.
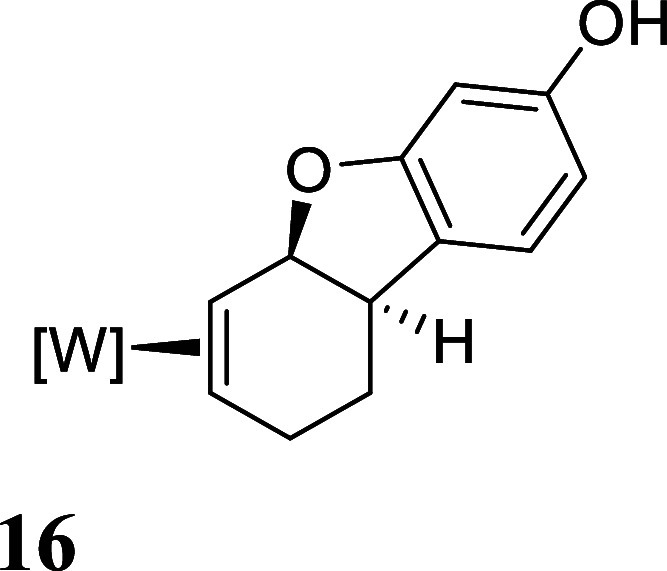


Used general procedure **2** with **13** (0.150 g, 0.179 mmol) and K­(t-BuO) (20% w/w in THF) (0.250 g, 0.446 mmol). Tan solid (0.0662 g, (53.8%)).

^1^H NMR (800 MHz, DMSO) δ: 8.09 (d, *J* = 2.1 Hz, 1H), 8.06 (d, *J* = 2.4 Hz, 1H), 8.04 (d, *J* = 2.0 Hz, 1H), 7.98 (d, *J* = 2.2 Hz, 1H), 7.91 (d, *J* = 2.4 Hz, 1H), 7.57 (d, *J* = 2.2 Hz, 1H), 6.81 (d, *J* = 7.8 Hz, 1H), 6.45 (t, *J* = 2.2 Hz, 1H), 6.34 (t, *J* = 2.2 Hz, 1H), 6.28 (t, *J* = 2.2 Hz, 1H), 6.08 (d, *J* = 11.0 Hz, 2H), 5.37 (d, *J* = 7.0 Hz, 1H), 3.00–2.95 (m, 1H), 2.82 (t, *J* = 11.5 Hz, 1H), 2.78–2.71 (m, 1H), 2.64–2.57 (m, 1H), 1.82 (dd, *J* = 12.1, 4.1 Hz, 1H), 1.27–1.19 (m, 2H), 1.18–1.05 (m, 11H). ^13^C NMR (201 MHz, DMSO) δ: 160.3, 143.0, 142.0, 140.8, 137.3, 136.9, 136.4, 136.1, 123.7, 107.9, 106.7, 106.3, 105.8, 97.7, 88.5 (d, *J* = 5.2 Hz), 52.9 (d, *J* = 9.9 Hz), 51.4, 40.8, 30.0, 26.9. ESI-HRMS (*m*/*z*): [M]^+^ calculated for C_24_H_31_BN_7_O_3_PW, 691.1828; found, 691.1780.
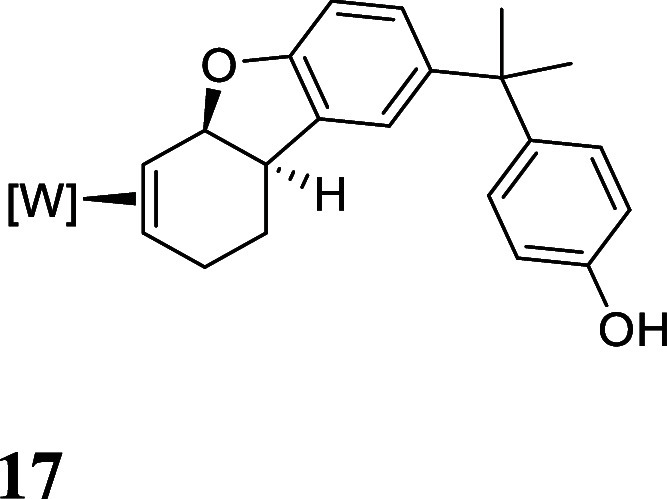


Used general procedure **2** with **11** (0.7820 g, 0.8151 mmol) and K­(t-BuO) (20% w/w in THF) (1.3845 g, 2.4977 mmol). Tan solid (0.6325 g, (96%)).

^1^H NMR (800 MHz, DMSO) δ: 8.09 (d, *J* = 2.1 Hz, 1H), 8.06 (d, *J* = 2.4 Hz, 1H), 8.05 (d, *J* = 2.0 Hz, 1H), 7.99 (d, *J* = 2.3 Hz, 1H), 7.91 (d, *J* = 2.5 Hz, 1H), 7.57 (d, *J* = 2.2 Hz, 1H), 7.08 (d, *J* = 2.1 Hz, 1H), 6.90 (dd, *J* = 8.2, 2.1 Hz, 1H), 6.60–6.55 (m, 2H), 6.45 (t, *J* = 2.1 Hz, 1H), 6.34 (t, *J* = 2.2 Hz, 1H), 6.29 (t, *J* = 2.2 Hz, 1H), 5.96–5.92 (m, 2H), 5.42 (d, *J* = 7.1 Hz, 1H), 3.10–3.06 (m, 1H), 2.82 (t, *J* = 11.5 Hz, 1H), 2.79–2.74 (m, 0H), 2.65–2.60 (m, 1H), 1.88–1.83 (m, 1H), 1.49 (d, *J* = 4.2 Hz, 5H), 1.28–1.24 (m, 1H), 1.24–1.20 (m, 1H), 1.11 (d, *J* = 8.3 Hz, 9H). ^13^C NMR (201 MHz, DMSO) δ: 169.8, 157.0, 144.6, 143.0, 142.0, 140.7, 136.9, 136.4, 136.2, 127.9, 127.5, 126.6, 125.3, 122.2, 117.9, 107.5, 106.8, 106.4, 105.8, 88.6 (d, *J* = 5.0 Hz), 52.3 (d, *J* = 10.0 Hz), 51.2, 41.5, 41.5, 31.6 (d, *J* = 8.5 Hz), 29.5, 26.8, 11.8 (d, *J* = 27.9 Hz). Confirmed by single-crystal X-ray diffraction (Figure 4).
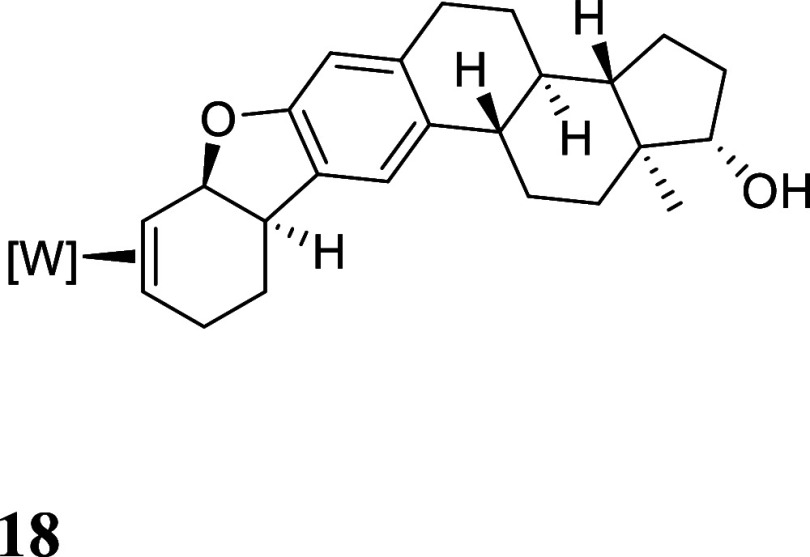


Used general procedure **1** with 8 (0.240 g, 0.232 mmol), NaBH_4_ (0.0527 g, 1.39 mmol), and HOTf (0.139 g, 0.930 mmol) in MeOH (3 mL). Tan solid (0.067 g, (29%)). Product carried onto next step without characterization. Used general procedure **2** (0.7834 g, 0.7806 mmol) and K­(t-BuO) (0.7 M in THF) (2 mL, 1 mmol). Tan solid (0.3147 g, (47%)).

#### For (R_W_,R,R,S)-**18**

^1^H NMR (600 MHz, CD3CN) δ: 8.16 (d, *J* = 2.0 Hz, 1H), 8.06 (d, *J* = 2.0 Hz, 1H), 7.89 (d, *J* = 2.3 Hz, 1H), 7.82 (d, *J* = 2.3 Hz, 1H), 7.77 (d, *J* = 2.4 Hz, 1H), 7.47 (d, *J* = 2.2 Hz, 1H), 7.19 (s, 1H), 6.46 (s, 1H), 6.40 (t, *J* = 2.2 Hz, 1H), 6.28 (t, *J* = 2.2 Hz, 1H), 6.24 (t, *J* = 2.2 Hz, 1H), 5.49 (d, *J* = 7.0 Hz, 1H), 3.60 (t, *J* = 8.6 Hz, 1H), 3.18 (dt, *J* = 9.3, 5.9 Hz, 1H), 2.82 (m, 5H), 2.68 (m, 1H), 2.34 (m, 1H), 2.17 (td, *J* = 11.2, 4.2 Hz, 1H), 2.03–1.82 (overlapping, 6H), 1.66 (m, 1H), 1.46–1.21 (overlapping, 7H), 1.14 (d, *J* = 8.3 Hz, 9H), 0.74 (s, 3H). Racemic composition confirmed by single-crystal X-ray diffraction.
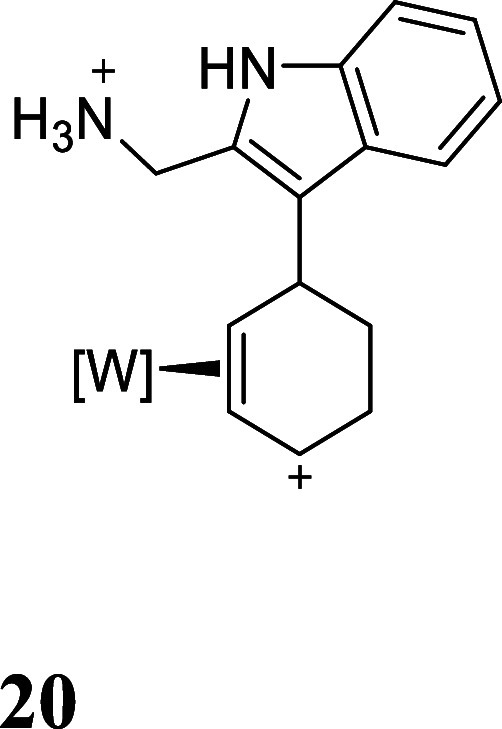


#### From

Used general procedure **2** with **9** (0.313 g, 0.343 mmol), NaBH4 (0.0784 g, 2.07 mmol) and HOTf (0.155 g, 1.04 mmol) in DME (2 mL). Tan solid (0.221 g, (73.2%))

^1^H NMR (400 MHz, CD_3_CN) δ: 9.80 (s, 1H), 8.43 (d, *J* = 2.3 Hz, 1H), 8.20 (d, *J* = 2.3 Hz, 1H), 8.00 (d, *J* = 2.4 Hz, 1H), 7.97 (dt, *J* = 2.5, 0.8 Hz, 1H), 7.88 (dd, *J* = 7.2, 2.5 Hz, 0H), 7.86–7.80 (m, 2H), 7.49 (dd, *J* = 8.2, 0.9 Hz, 1H), 7.28 (ddd, *J* = 8.2, 7.0, 1.1 Hz, 1H), 7.22–7.14 (m, 1H), 6.91 (s, 3H), 6.62–6.55 (m, 1H), 6.54 (t, *J* = 2.4 Hz, 1H), 6.48 (t, *J* = 2.3 Hz, 1H), 6.38 (t, *J* = 2.4 Hz, 1H), 5.54 (t, *J* = 7.3 Hz, 1H), 4.54 (d, *J* = 7.0 Hz, 1H), 4.50 (d, *J* = 6.9 Hz, 1H), 4.43–4.33 (m, 1H), 4.16 (dd, *J* = 11.6, 6.2 Hz, 1H), 3.70–3.56 (m, 1H), 3.44–3.36 (m, 1H), 1.93–1.86 (m, 1H), 1.70–1.58 (m, 1H), 1.00 (d, *J* = 9.8 Hz, 9H). ^13^C NMR (101 MHz, CD_3_CN) δ: 149.2, 146.2, 143.4, 139.6, 139.6 (d, *J* = 3.3 Hz), 137.9, 133.4, 126.2, 125.4, 124.3, 122.5, 121.5, 120.8, 112.9, 109.6, 109.0, 108.1, 106.3 (d, *J* = 3.5 Hz), 76.1 (d, *J* = 12.2 Hz), 36.4, 35.5, 29.5, 26.1, 13.8 (d, *J* = 32.7 Hz). ESI-HRMS (*m*/*z*): [M]^+^ calculated for C_27_H_36_BN_9_O_2_PW^+^, 728.2372; found, 728.2376.
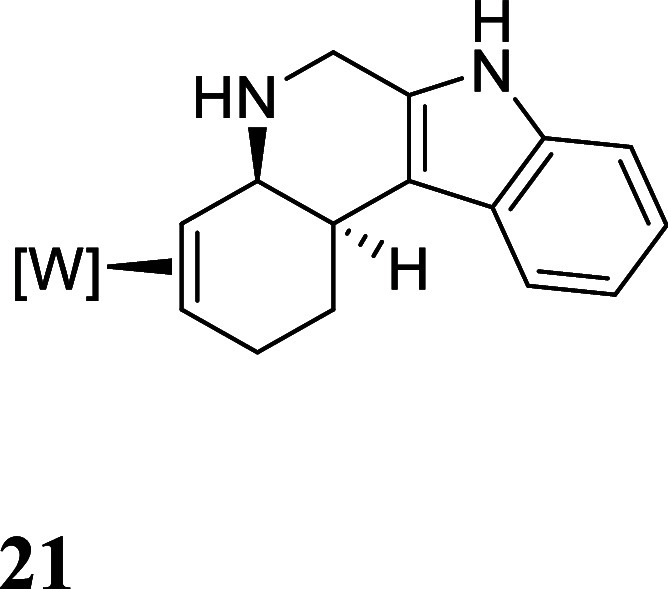


To a 34 mL test tube was added **20** (0.336 g, 0.327 mmol) and THF (5 mL). To a separate 15 mL test tube was added LiHMDS (1.0 M in THF) (3 mL, 2 mmol). Both solutions were chilled to −60 °C for 10 min before adding the solution of LiHMDS to **20**. The reaction mixture was allowed to stir at −60 °C for 15 min before diluting with DCM (5 mL) and washing with saturated Na_2_CO_3_ (2 × 5 mL). The organic layer was dried over anhydrous Na_2_SO_4_ and concentrated in vacuo. The film was dissolved in minimal DCM and added to 200 mL of stirring pentanes to precipitate a tan solid which was collected on a 30 mL medium-porosity fritted funnel and dried. (0.145 g, (60.9%))

^1^H NMR (400 MHz, CD3CN) δ: 8.87 (s, 1H), 7.49–7.42 (m, 2H), 7.32 (dt, *J* = 8.0, 1.0 Hz, 1H), 7.04 (ddd, *J* = 8.1, 7.0, 1.3 Hz, 1H), 6.98 (ddd, *J* = 8.1, 7.1, 1.2 Hz, 1H), 6.39 (t, *J* = 2.2 Hz, 1H), 6.30 (t, *J* = 2.2 Hz, 1H), 6.22 (t, *J* = 2.2 Hz, 1H), 4.12–4.04 (m, 2H), 4.03–3.97 (m, 1H), 3.39–3.30 (m, 1H), 3.15 (d, *J* = 12.5 Hz, 0H), 2.94–2.84 (m, 1H), 2.56 (t, *J* = 11.4 Hz, 1H), 2.01–1.97 (m, 0H), 1.56–1.45 (m, 1H), 1.12 (d, *J* = 8.3 Hz, 9H). ^13^C NMR (101 MHz, CD_3_CN) δ: 144.0, 143.0, 141.7, 137.8, 137.3, 137.0, 137.0, 135.2, 128.6, 121.4, 119.5, 111.8, 107.6, 106.9, 106.5, 60.6 (d, *J* = 9.7 Hz), 60.3 (d, *J* = 5.0 Hz), 52.3, 44.5, 33.5, 31.7, 29.6, 13.2 (d, *J* = 27.7 Hz). ESI-HRMS (*m*/*z*): [M + H]^+^ calculated for C_27_H_35_BN_9_O_2_PW, 728.2226; found, 728.2377 corresponding to C_27_H_36_BN_9_O_2_PW^+^ (**26**).
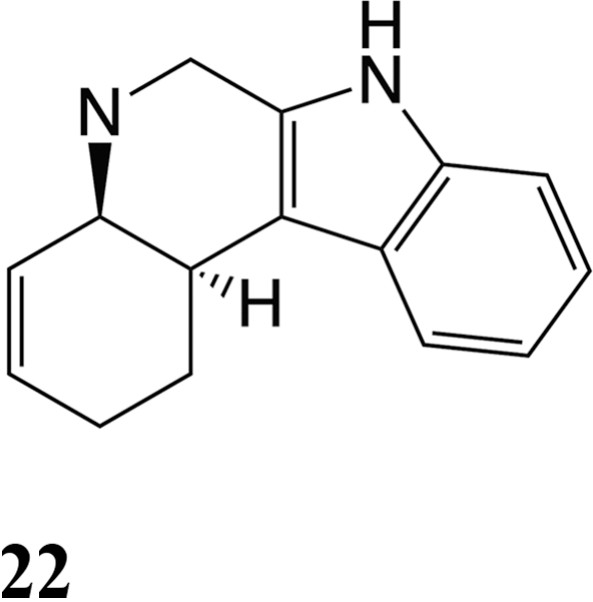


To a 4-dram vial was charged with **21** (0.173 g, 0.238 mmol) a stir pea and MeCN (∼4 mL). To this vial was added Br_2_ (0.0452 g, 0.286 mmol, 1.0 M in DCE) and the reaction was allowed to stir for 2 min. The solution was then quenched with a solution of Na_2_S_2_O_3_ (∼2 mL, 10% w/w). The solution was then basified to a pH of ∼12 with a NaOH solution (1.0 M) and extracted with DCM (2 × 5 mL). The resulting organic layer was concentrated in vacuo to yield a light-yellow oil (9.2 mg (17.2%)).

^1^H NMR (600 MHz, CDCl_3_) δ: 7.83 (s, 1H), 7.57 (d, *J* = 7.7 Hz, 1H), 7.31 (d, *J* = 8.0 Hz, 1H), 7.15 (t, *J* = 1.3 Hz, 1H), 7.10 (t, *J* = 1.1 Hz, 1H), 5.99–5.94 (m, 1H), 5.94–5.88 (m, 1H), 4.11–3.95 (m, 2H), 3.43 (d, *J* = 5.2 Hz, 1H), 3.02–2.96 (m, 1H), 2.19 (ddt, *J* = 13.5, 5.5, 2.5 Hz, 2H), 2.17–2.08 (m, 1H), 1.58–1.48 (m, 1H). ^13^C NMR (151 MHz, CDCl_3_) δ: 135.9, 133.0, 130.7, 128.5, 127.1, 121.4, 119.3, 118.0, 113.3, 110.8 (d, *J* = 7.7 Hz), 51.2, 42.9, 32.2, 25.5, 25.2. APCI-HRMS (*m*/*z*): [M + H]^+^ calculated for C_15_H_17_N_2_^+^, 225.1383; found, 225.1387 corresponding to C_15_H_17_N_2_^+^.
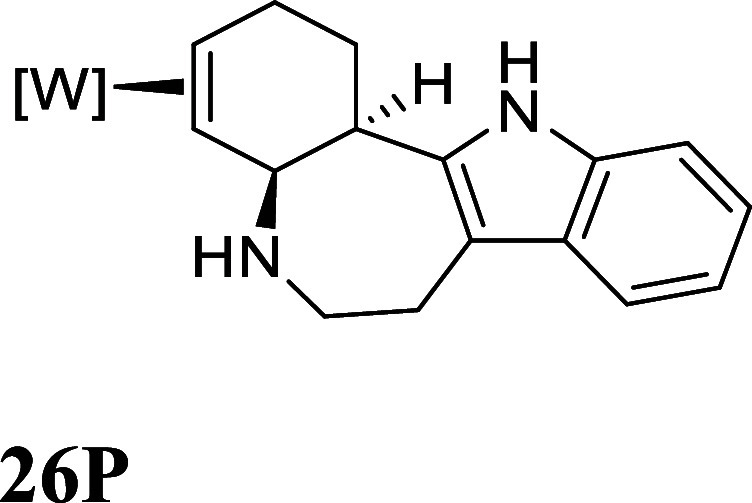


To a 30 mL test tube was added **24P** (0.9359 g, 0.8987 mmol) and THF (5 mL). To a separate 15 mL test tube was added LiHMDS (1.0 M in THF) (3 mL, 2 mmol). Both solutions were chilled to −60 °C for 10 min before adding the solution of LiHMDS to **24P**. The reaction mixture was allowed to stir at −60 °C for 15 min before diluting with DCM (5 mL) and washing with saturated Na_2_CO_3_ (2 × 5 mL). The organic layer was dried over anhydrous Na_2_SO_4_ and concentrated in vacuo. Film dissolved in minimal DCM and added to 300 mL of stirring pentanes to produce a precipitate which was collected on a 30 mL medium-porosity fritted funnel. White solid (0.4026 g, (60%)).

^1^H NMR (600 MHz, CD_3_CN) δ: 9.18 (s, 1H), 8.14 (d, *J* = 2.0 Hz, 1H), 8.10 (d, *J* = 2.1 Hz, 1H), 7.90 (d, *J* = 2.4 Hz, 1H), 7.85 (d, *J* = 2.3 Hz, 1H), 7.70 (d, *J* = 2.4 Hz, 1H), 7.57 (d, *J* = 2.3 Hz, 1H), 7.44 (d, *J* = 7.9 Hz, 1H), 7.29 (d, *J* = 8.0 Hz, 1H), 7.03–6.98 (m, 1H), 6.98–6.93 (m, 1H), 6.42 (t, *J* = 2.3 Hz, 1H), 6.30 (t, *J* = 2.3 Hz, 1H), 6.17 (t, *J* = 2.3 Hz, 1H), 4.51 (d, *J* = 7.1 Hz, 1H), 3.59 (t, *J* = 10.7 Hz, 1H), 2.96–2.87 (m, 2H), 2.87–2.81 (m, 2H), 2.49–2.40 (m, 2H), 2.15–2.10 (m, 1H), 1.93–1.87 (m, 1H), 1.85–1.77 (m, 1H), 1.20 (d, *J* = 9.2 Hz, 9H). ^13^C NMR (151 MHz, CD_3_CN) δ: 144.6, 143.4, 142.3, 138.4, 137.7, 137.5, 121.3, 119.5, 118.8, 111.6, 107.8, 107.2, 106.6, 64.1, 58.7 (d, *J* = 6.7 Hz), 36.7, 33.5, 30.8, 28.8, 12.4 (d, *J* = 29.1 Hz).
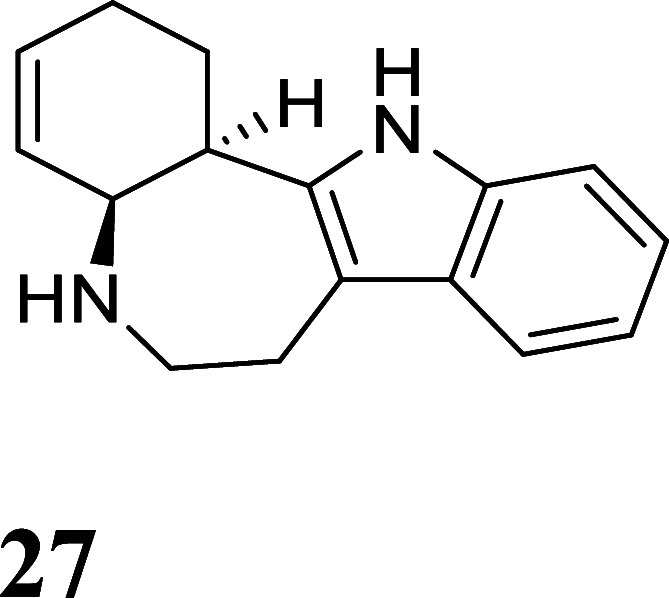


To a 15 mL test tube was added **26P** (0.1102 g, 0.1485 mmol) and acetone (3 mL). This solution was chilled to −30 °C for 5 min before slowly adding a chilled (−30 °C) solution of NOPF_6_ (0.0443 g, 0.2532 mmol) in MeCN (1 mL). After 5 min, reaction removed from the glovebox, diluting with DCM (5 mL) and washing with saturated Na_2_CO_3_ (3 × 5 mL). The aqueous layers were combined and back-extracted with DCM (5 mL). The organic layers were combined, dried over Na_2_SO_4_, and evaporated to a film. This was dissolved in minimal CH_2_Cl_2_ and added to a 1:1 solution of hexanes and Et_2_O (200 mL). A precipitate was collected on a 15 mL medium-porosity fritted funnel and washed with Et_2_O (3 × 10 mL). The filtrate was evaporated to dryness, dissolved in minimal CH_2_Cl_2_, and purified via flash chromatography on basic alumina (EtOAc/hexanes, ∼ 80%). Yellow oil (4.4 mg (12%)).

^1^H NMR (800 MHz, CD_2_Cl_2_) δ: 7.82 (s, 1H, N-H (indole)), 7.42 (d, *J* = 7.7 Hz, 1H, H16), 7.26 (d, *J* = 7.9 Hz, 1H, H13), 7.08 (t, *J* = 7.4 Hz, 1H, H14), 7.03 (t, *J* = 7.4 Hz, 1H, H15), 5.83 (dd, *J* = 11.4, 5.1 Hz, 1H, H2), 5.79 (t, *J* = 6.6 Hz, 1H, H1), 3.48 (t, *J* = 4.3 Hz, 1H, H3), 3.41 (dt, *J* = 13.2, 3.6 Hz, 1H, H10x), 2.95 (overlapping, 2H, H4 and H9x), 2.83 (t, *J* = 12.7 Hz, 1H, H10y), 2.73 (ddd, *J* = 15.7, 12.1, 3.9 Hz, 1H, H9y), 2.17 (overlapping, 2H, H6), 2.12 (qd, *J* = 12.7, 6.1 Hz, 1H, H5x), 1.71 (dd, *J* = 13.5, 5.3 Hz, 1H, H5y). ^13^C NMR (201 MHz, CD_2_Cl_2_) δ: 139.3 (C7), 135.6 (C12), 130.1 (C2), 129.9 (C11), 129.0 (C1), 121.5 (C14), 119.4 (C15), 118.3 (C16), 112.4 (C8), 110.5 (C13), 56.4 (C3), 50.5 (C10), 43.2 (C4), 29.1 (C9), 26.9 (C6), 24.8 (C5).

## Supplementary Material



## Data Availability

NMR data: 10.5281/zenodo.17466809.

## Notes

^1^H and ^13^C NMR spectra of selected compounds, and crystallographic information for compounds **10**, **14**, **15**, **17**, **18**, and **26P** (PDF)
