# Supplementary material for: Formation of Heteropolycyclic Frameworks via the Dearomatization of a Dihapto-Coordinated Anisole
Source: Organometallics. 2025 Nov 20;44(23):2796–806. doi: 10.1021/acs.organomet.5c00410 (PMC12690591; doi:10.1021/acs.organomet.5c00410)
Supplement: Supplementary file 1 [file om5c00410_si_001.pdf]

Supplementary Information

Formation of heteropolycyclic frameworks via the dearomatization of a dihapto-coordinated anisole

Authors: Mason R. Ortiz[1‡], Justin T. Weatherford-Pratt[1‡], Jeremy M. Bloch[1], Diane A. Dickie[1], and W. Dean Harman [1*]

Affiliation: [1]Department of Chemistry, University of Virginia; Charlottesville, VA 22904 U.S.A.

‡ Denotes co-first author

*Corresponding author. Email: wdh5z@virginia.edu

Table of Contents

NMR Spectra 2

Figure S-1. Compound 9 ^{1}H NMR (400 MHz, CD_2Cl_2, δ, 25 °C) & ^{13}C NMR (101 MHz, CD_2Cl_2, δ, 25 °C) 2

Figure S-2. Compound 10 ^{1}H NMR (800 MHz, MeCN, δ, 25 °C) & ^{13}C NMR (201 MHz, MeCN, δ, 25 °C) 3

Figure S-3. Compound 11 ^{1}H NMR (800 MHz, Acetone δ, 25 °C) & ^{13}C NMR (201 MHz, Acetone, δ, 25 °C) 4

Figure S-4. Compound 12 ^{1}H NMR (800 MHz, MeCN δ, 25 °C) & ^{13}C NMR (201 MHz, MeCN, δ, 25 °C) 5

Figure S-5. Compound 13 ^{1}H NMR (400 MHz, (CD_2Cl_2, δ, 25 °C) & ^{13}C NMR (101 MHz, CD_2Cl_2, δ, 25 °C) 6

Figure S-6. Compound 14 ^{1}H NMR (800 MHz, MeCN, δ, 25 °C) & ^{13}C NMR (201 MHz, MeCN, δ, 25 °C) 7

Figure S-7. Compound 15 ^{1}H NMR (800 MHz, DMSO, δ, 25 °C) & ^{13}C NMR (201 MHz, DMSO, δ, 25 °C) 8

Figure S-8. Compound 16 ^{1}H NMR (800 MHz, DMSO, δ, 25 °C) & ^{13}C NMR (201 MHz, DMSO, δ, 25 °C) 9

Figure S-9. Compound 17 ^{1}H NMR (800 MHz, DMSO, δ, 25 °C) & ^{13}C NMR (201 MHz, DMSO, δ, 25 °C) 10

Figure S-10. Compound 18 ^{1}H NMR (600 MHz, CD_2Cl_2, δ, 25 °C) 11

Figure S-11. Compound 18, Methyl group region for 1:1 and 9:1 mixture of (R, R, R, S)-18 and (S, S, S, S)-18 (600 MHz, CD3CN). 11

Figure S-12. Compound 20 ^{1}H NMR (400 MHz, CD_3CN, δ, 25 °C) & ^{13}C NMR (101 MHz, CD_3CN, δ, 25 °C) 12

Figure S-13. Compound 21 ^{1}H NMR (400 MHz, CD_3CN, δ, 25 °C) & ^{13}C NMR (101 MHz, CD_3CN, δ, 25 °C) 13

Figure S-14. Compound 22 ^{1}H NMR (600 MHz, $CDCl_3$, δ, 25 °C) & ^{13}C NMR (151 MHz, $CDCl_3$, δ, 25 °C) 14

Figure S-15. Compound 26P ^{1}H NMR (800 MHz, CD_3CN, δ, 25 °C) & ^{13}C NMR (151 MHz, $CDCl_3$, δ, 25 °C) 15

Figure S-16. Compound 27 ^{1}H NMR (800 MHz, CD_2Cl_2, δ, 25 °C) & ^{13}C NMR (201 MHz, CD_2Cl_2, δ, 25 °C). 16

Crystallographic Data 17

Table S-1. Crystal data for **10**, **14**, **15**, **17**, **18**, and **26P**. 17

Experimental Details for SI(1) to SI(4) 18

Figure S-17. Compound SI(2) ^{1}H NMR (600 MHz, CD_3CN, δ, 25 °C) & ^{13}C NMR (151 MHz, CD_3CN, δ, 25 °C) .. 22

Figure S-18. Compound SI(3) ^{1}H NMR (800 MHz, CD_3CN, δ, 25 °C) & ^{13}C NMR (201 MHz, CD_3CN, δ, 25 °C) .. 23

Figure S-19. Compound SI(4) ^{1}H NMR (800 MHz, CD_3CN, δ, 25 °C) & ^{13}C NMR (201 MHz, CD_3CN, δ, 25 °C) .. 24

Supplementary References...25

NMR Spectra

Figure S-1. Compound 9 ^{1}H NMR (400 MHz, CD_2Cl_2, δ, 25 °C) & ^{13}C NMR (101 MHz, CD_2Cl_2, δ, 25 °C)

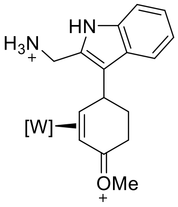

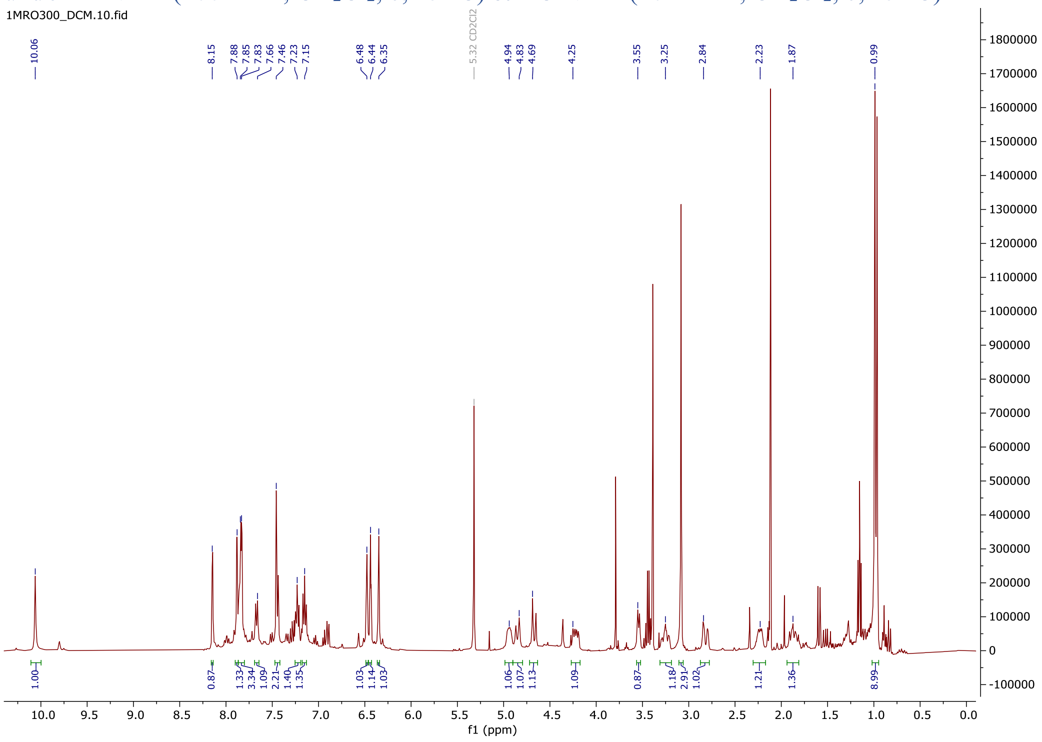

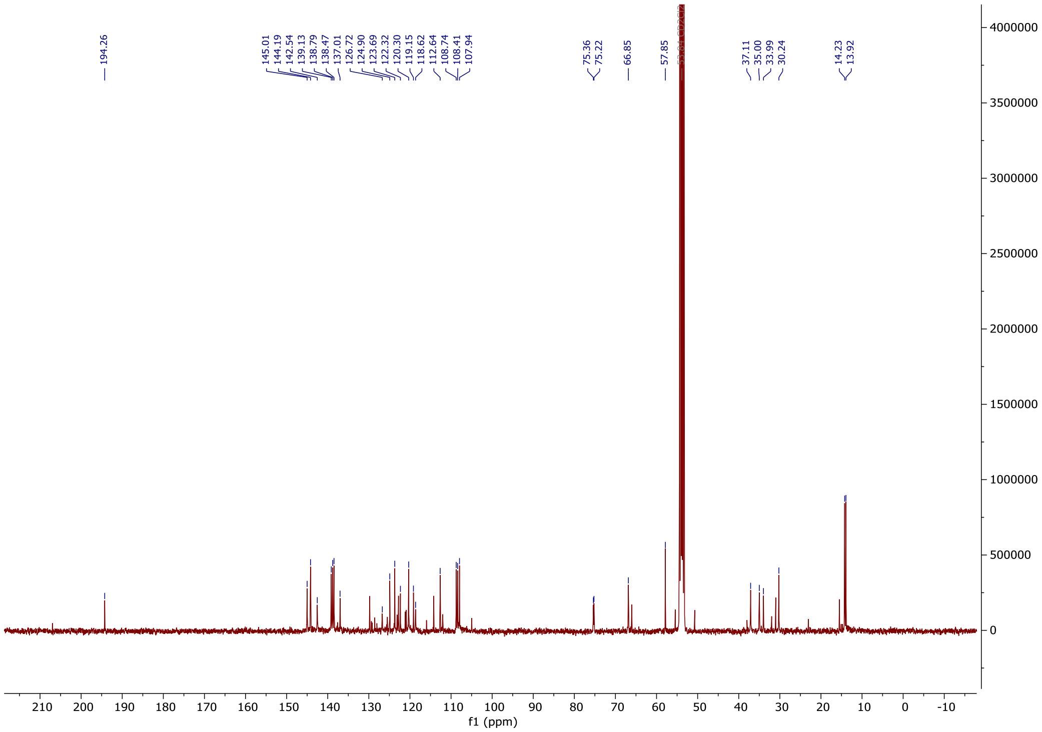

Figure S-2. Compound 10 ^{1}H NMR (800 MHz, MeCN, δ, 25 °C) & ^{13}C NMR (201 MHz, MeCN, δ, 25 °C)

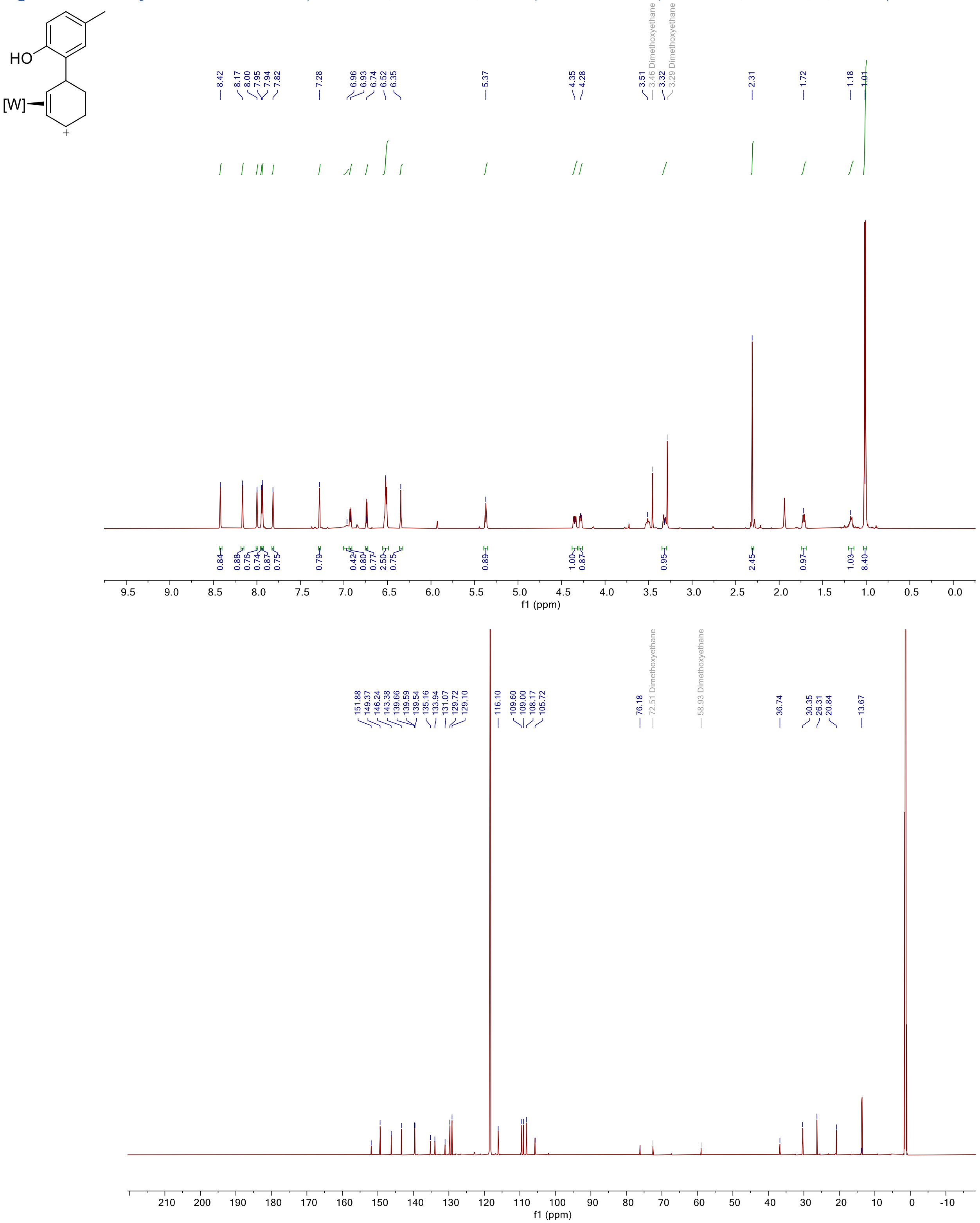

Figure S-3. Compound 11 ^{1}H NMR (800 MHz, Acetone δ, 25 °C) & ^{13}C NMR (201 MHz, Acetone, δ, 25 °C)

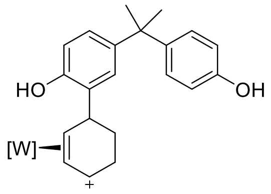

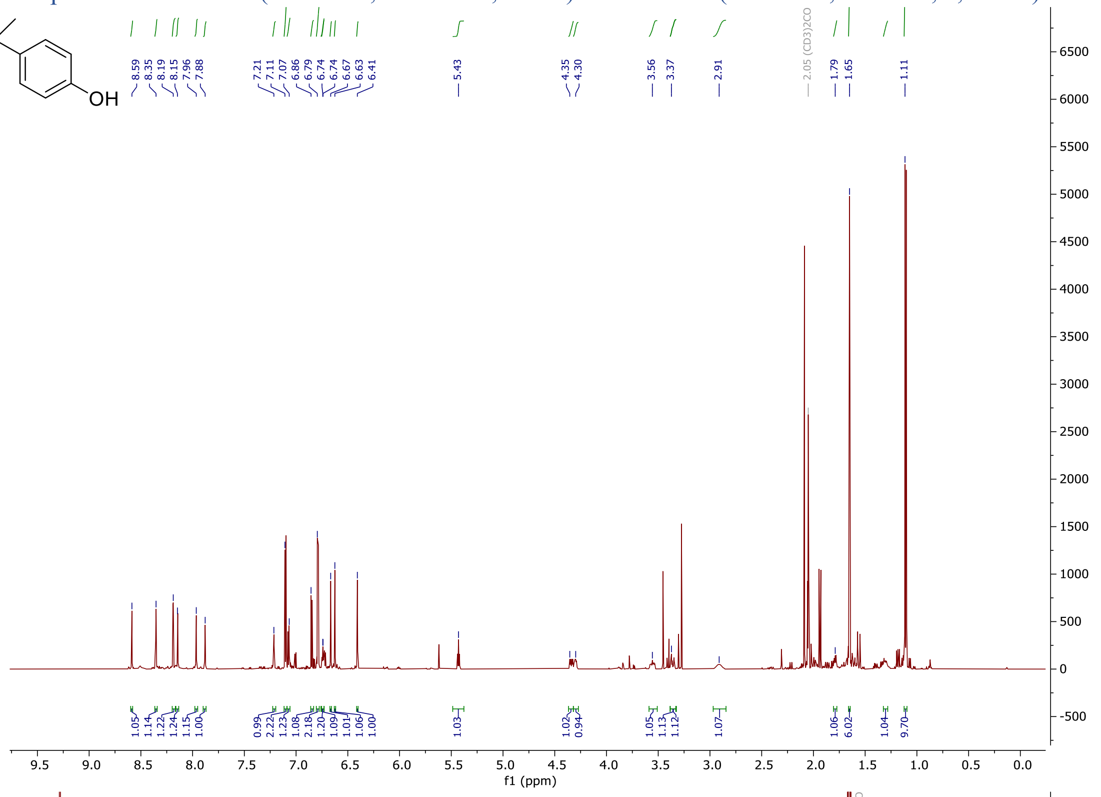

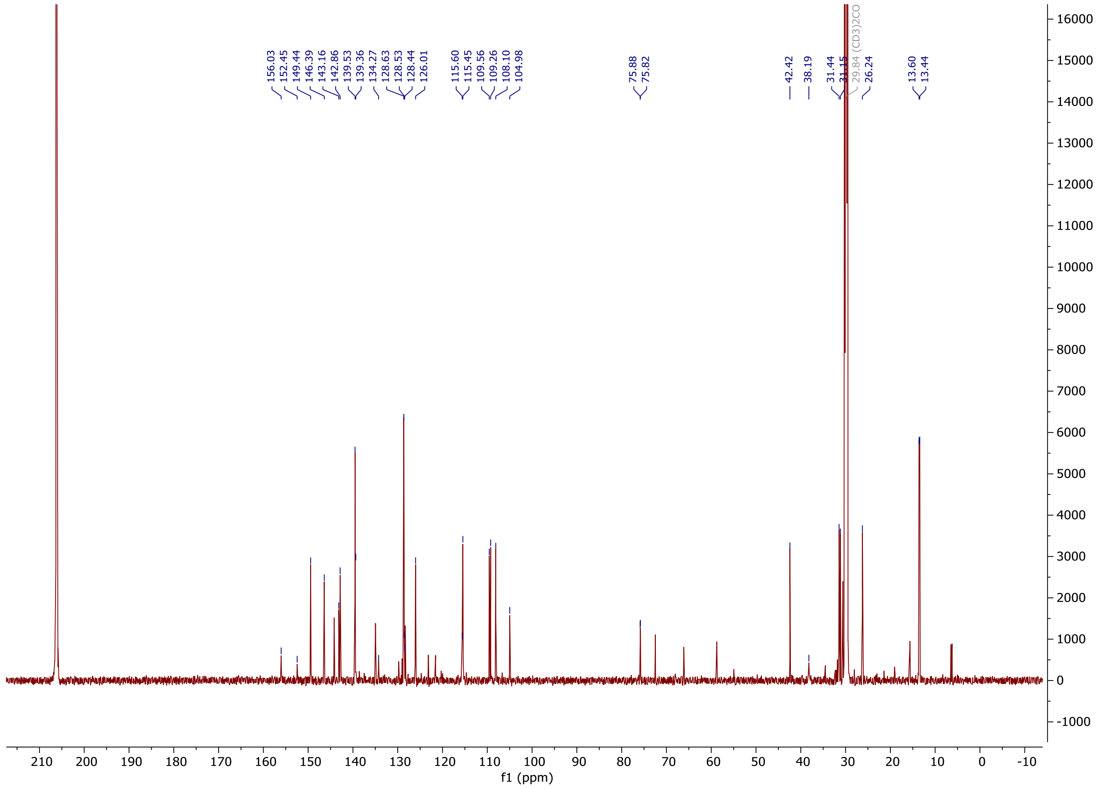

Figure S-4. Compound 12 ^{1}H NMR (800 MHz, MeCN δ, 25 °C) & ^{13}C NMR (201 MHz, MeCN, δ, 25 °C)

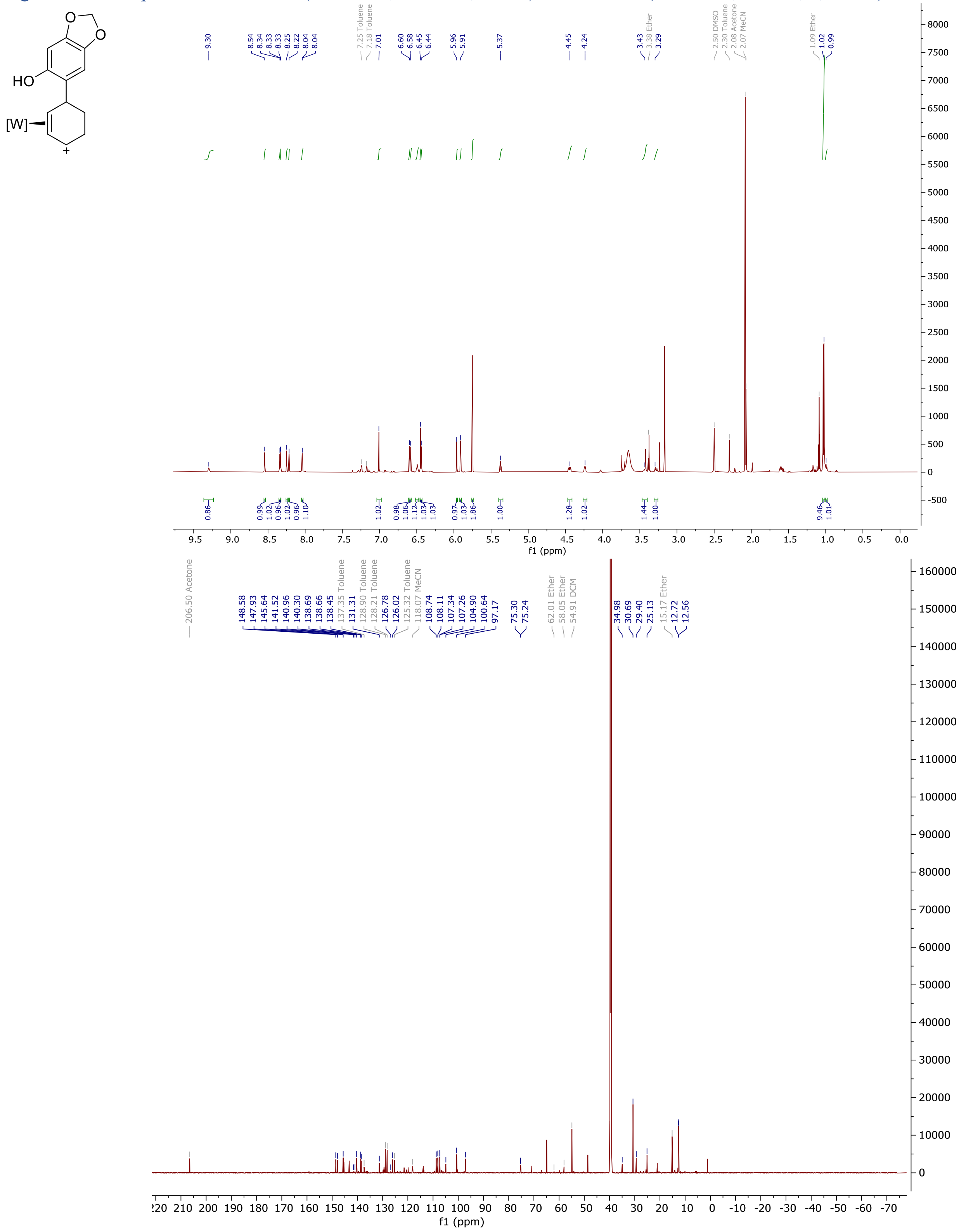

Figure S-5. Compound 13 ^{1}H NMR (400 MHz, (CD_2Cl_2, δ, 25 °C) & ^{13}C NMR (101 MHz, CD_2Cl_2, δ, 25 °C)

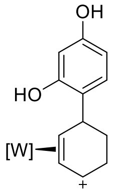

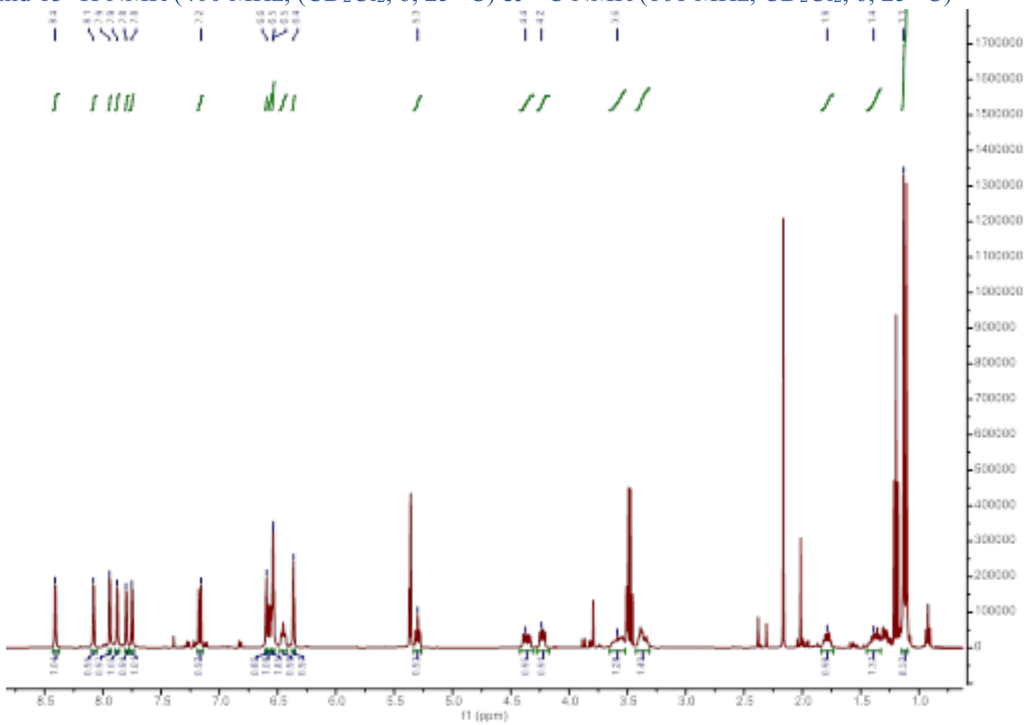

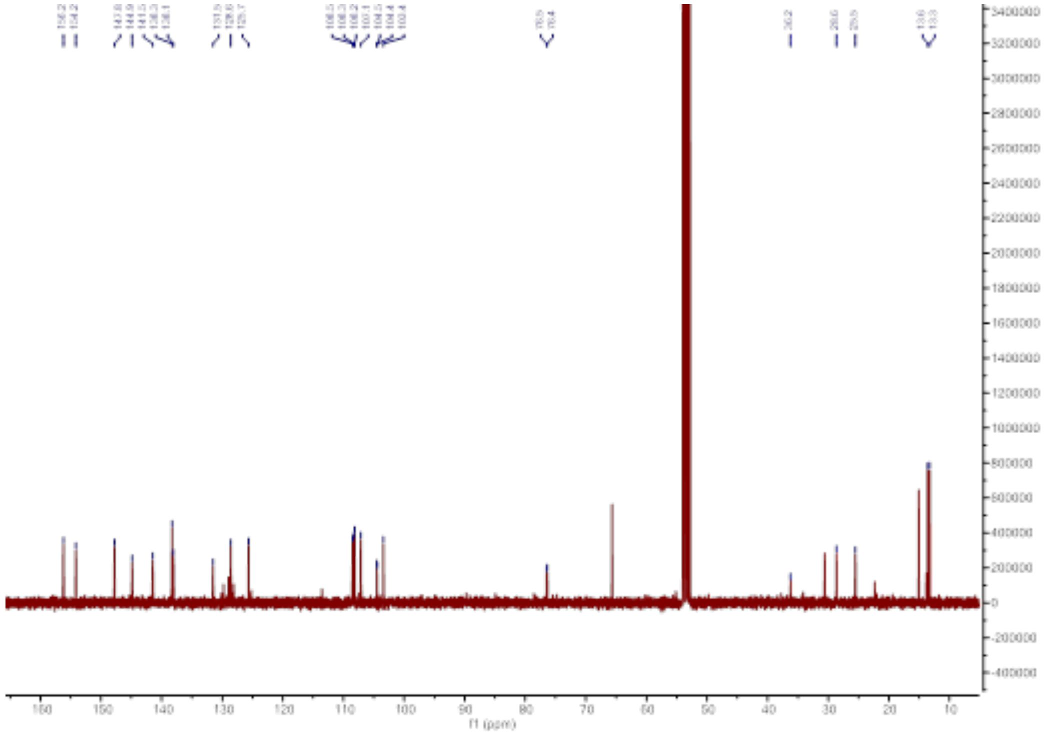

Figure S-6. Compound 14 ^{1}H NMR (800 MHz, MeCN, δ, 25 °C) & ^{13}C NMR (201 MHz, MeCN, δ, 25 °C)

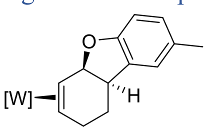

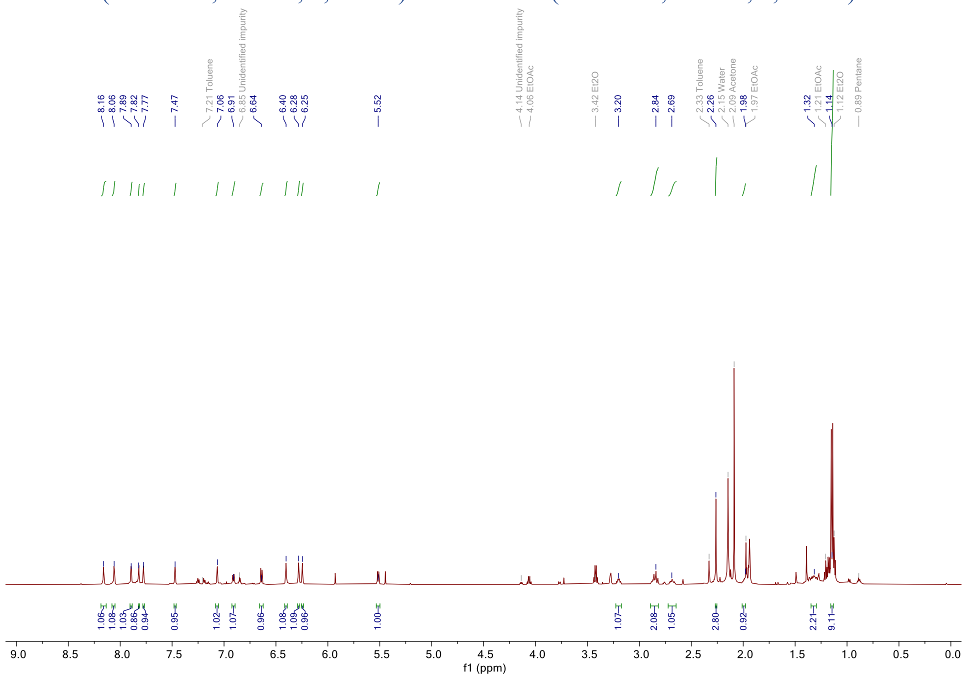

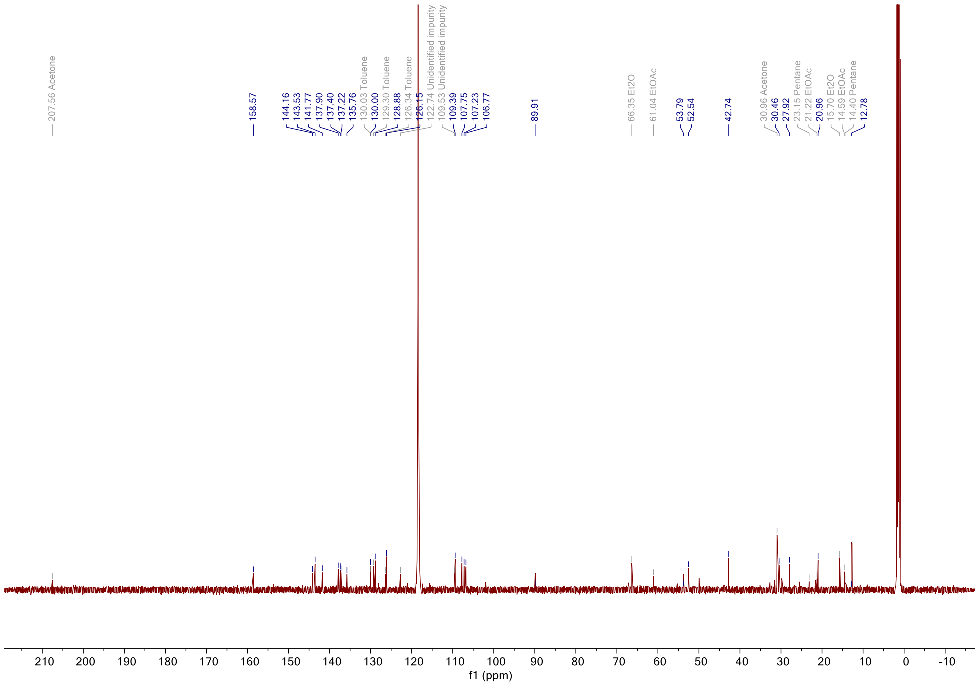

Figure S-7. Compound 15 ^{1}H NMR (800 MHz, DMSO, δ, 25 °C) & ^{13}C NMR (201 MHz, DMSO, δ, 25 °C)

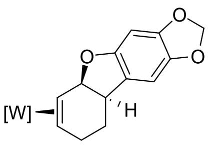

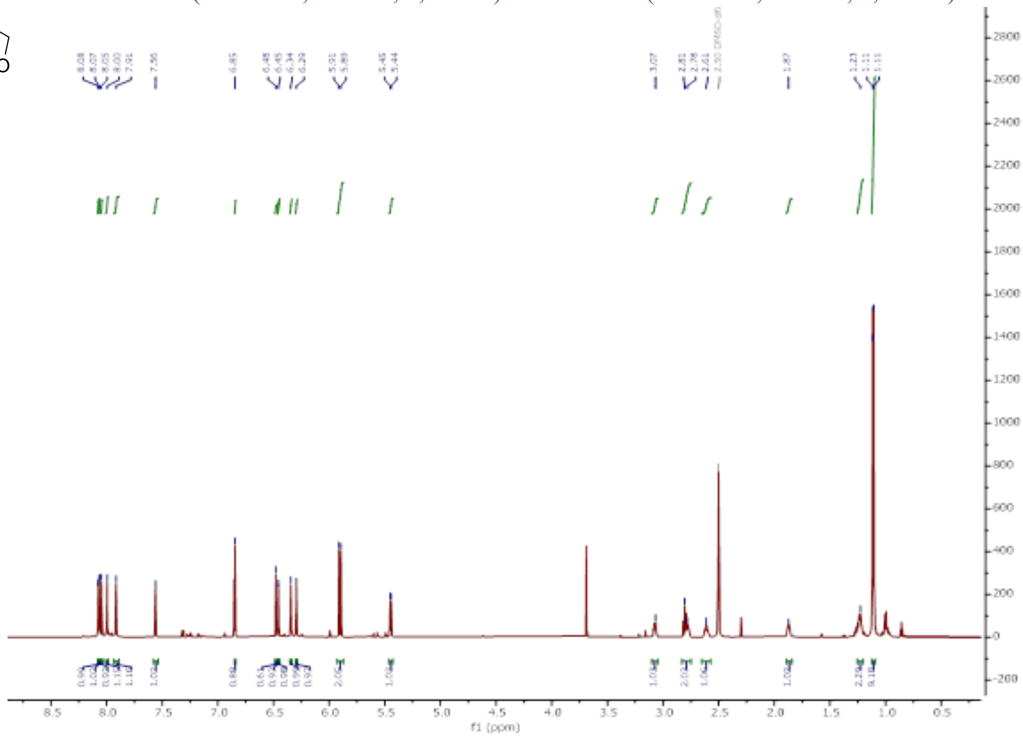

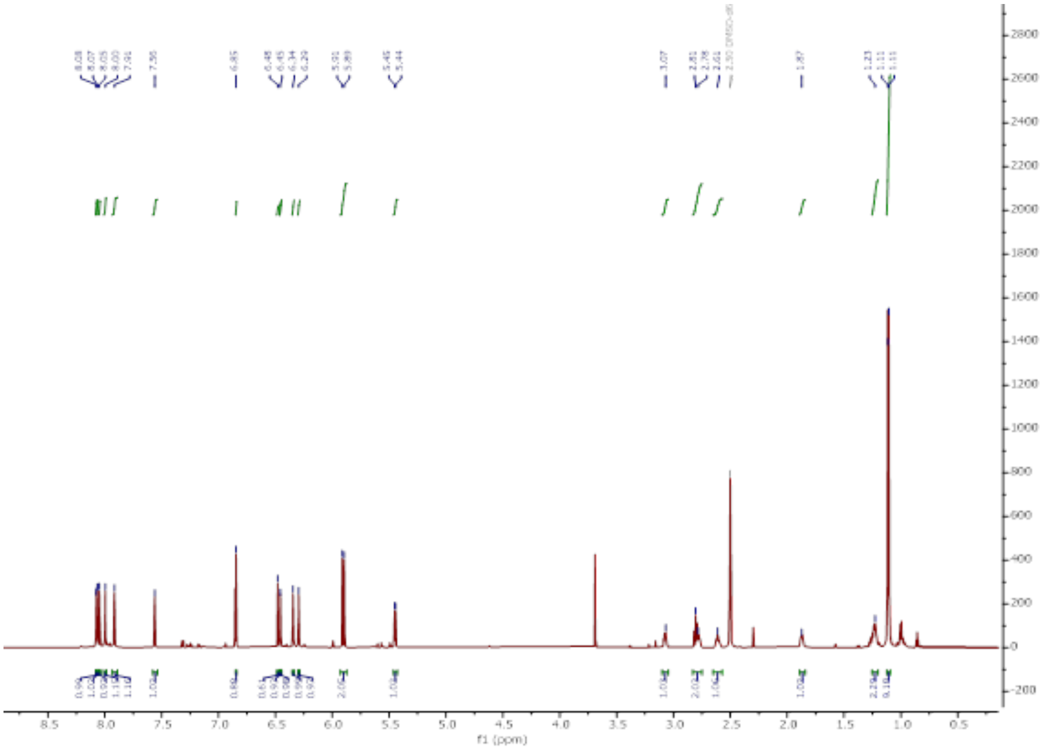

Figure S-8. Compound 16 ^{1}H NMR (800 MHz, DMSO, δ, 25 °C) & ^{13}C NMR (201 MHz, DMSO, δ, 25 °C)

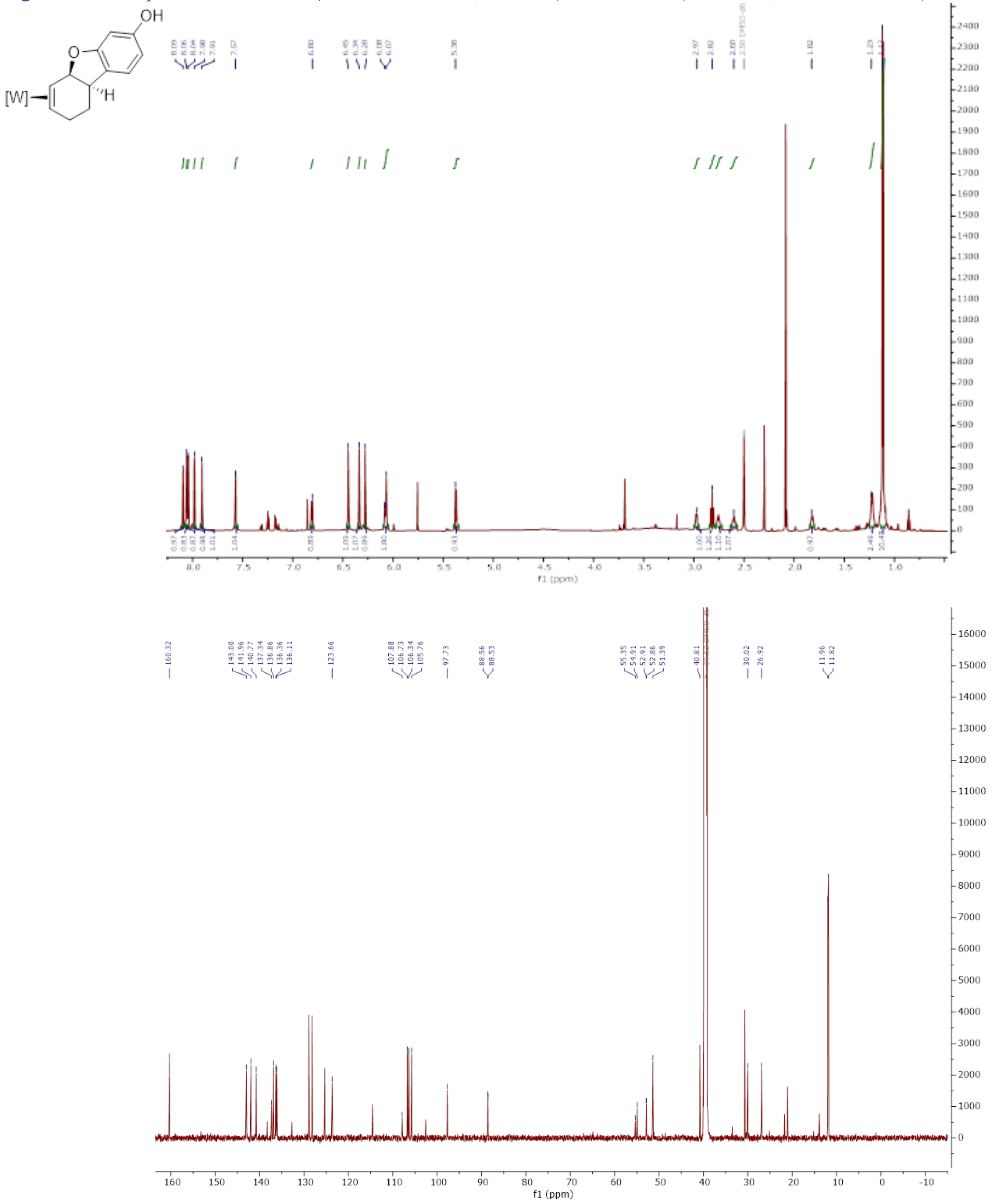

Figure S-9. Compound 17 ^{1}H NMR (800 MHz, DMSO, δ, 25 °C) & ^{13}C NMR (201 MHz, DMSO, δ, 25 °C)

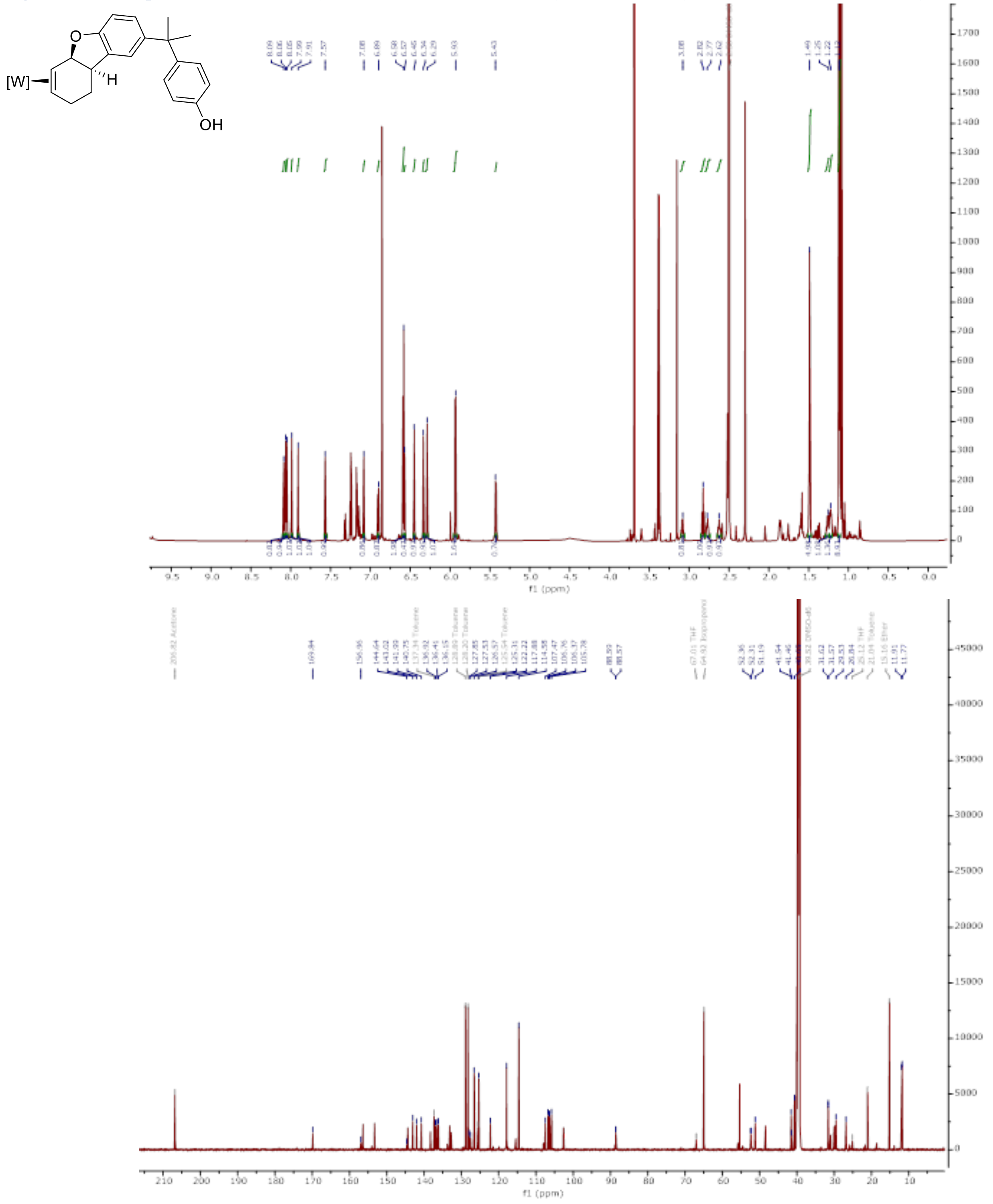

Figure S-10. Compound 18 ^{1}H NMR (600 MHz, CD_2Cl_2, δ, 25 °C)

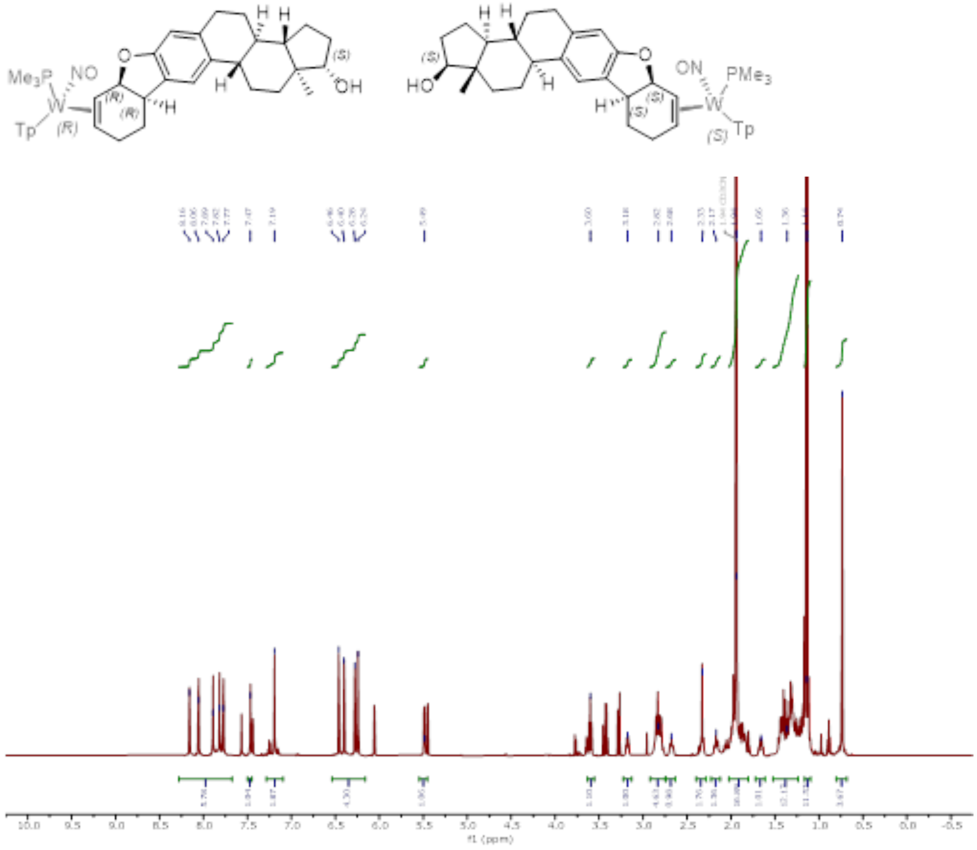

Figure S-11. Compound 18, Methyl group region for 1:1 and 9:1 mixture of (R, R, R, S)-18 and (S, S, S, S)-18 (600 MHz, CD3CN).

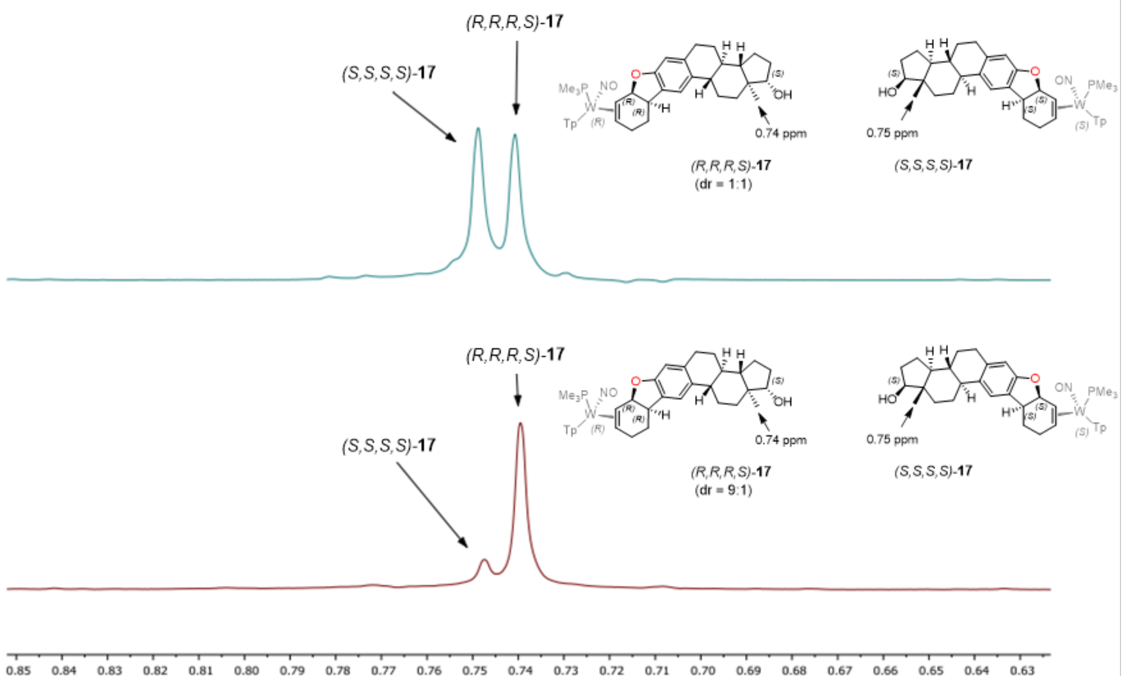

Figure S-12. Compound 20 ^{1}H NMR (400 MHz, CD_3CN, δ, 25 °C) & ^{13}C NMR (101 MHz, CD_3CN, δ, 25 °C)

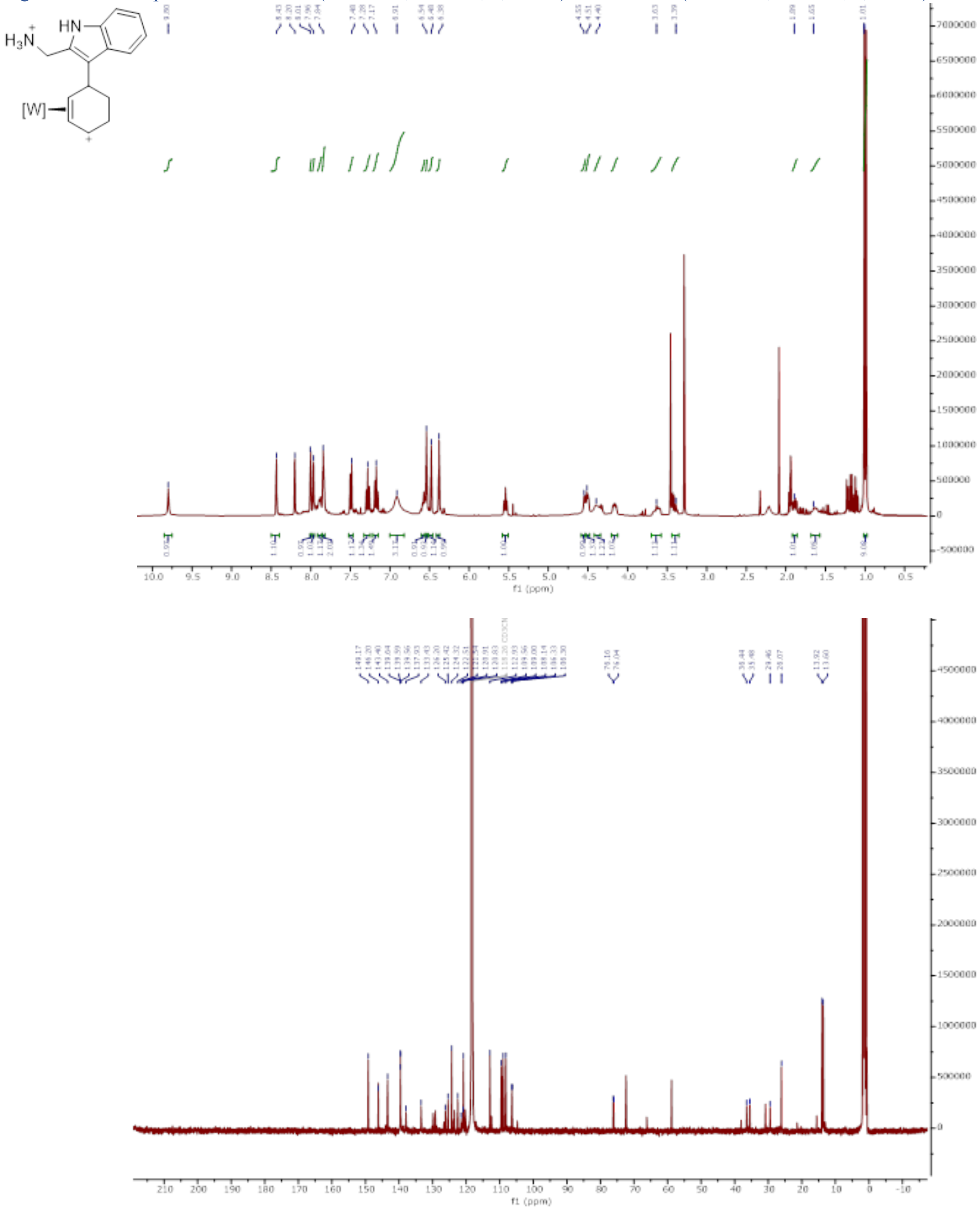

Figure S-13. Compound 21 ^{1}H NMR (400 MHz, CD_3CN, δ, 25 °C) & ^{13}C NMR (101 MHz, CD_3CN, δ, 25 °C)

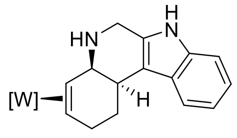

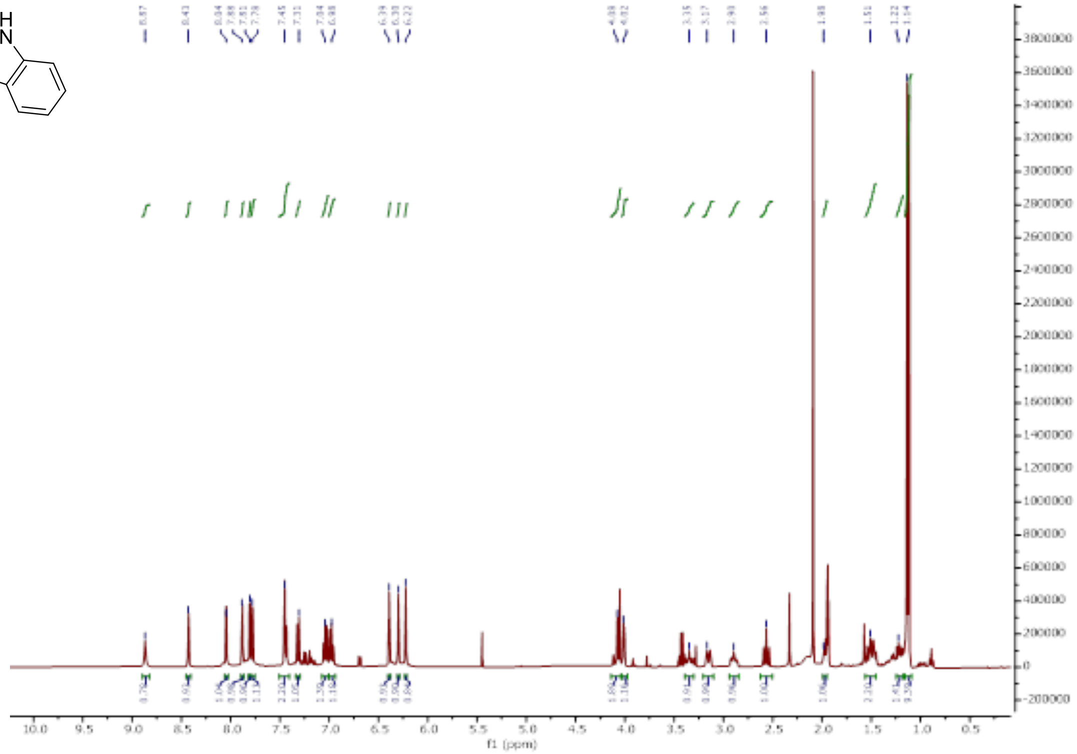

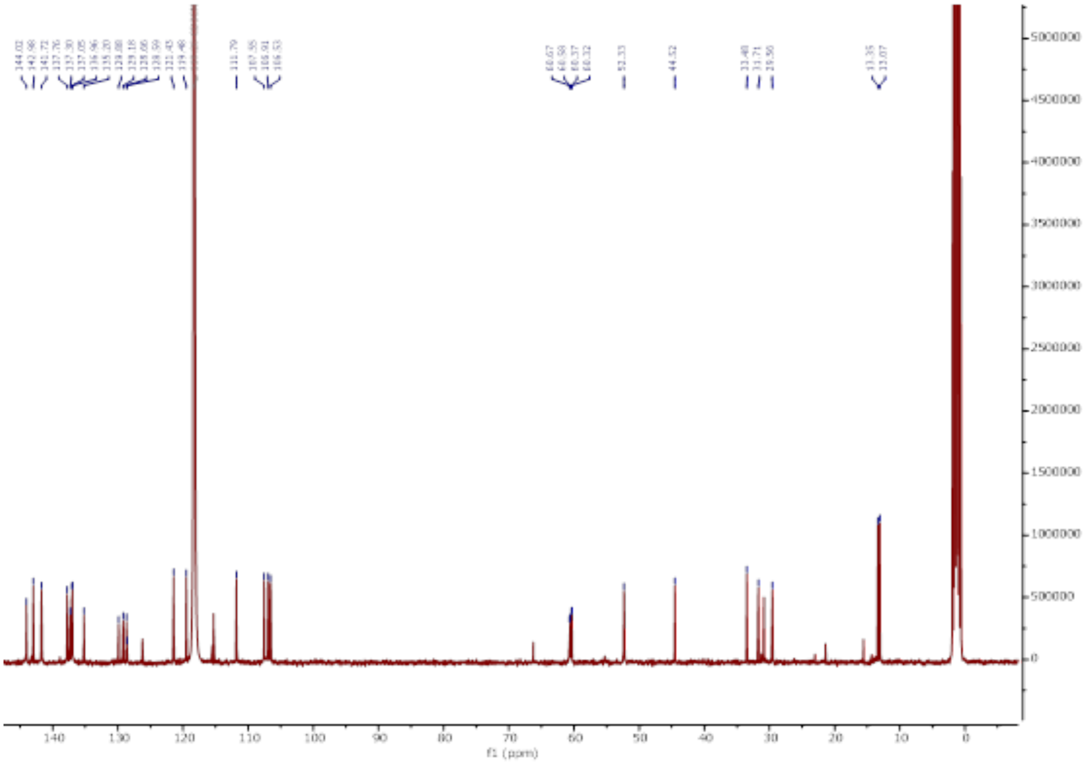

Figure S-14. Compound 22 ^{1}H NMR (600 MHz, $CDCl_3$, δ, 25 °C) & ^{13}C NMR (151 MHz, $CDCl_3$, δ, 25 °C)

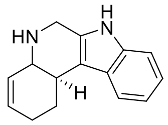

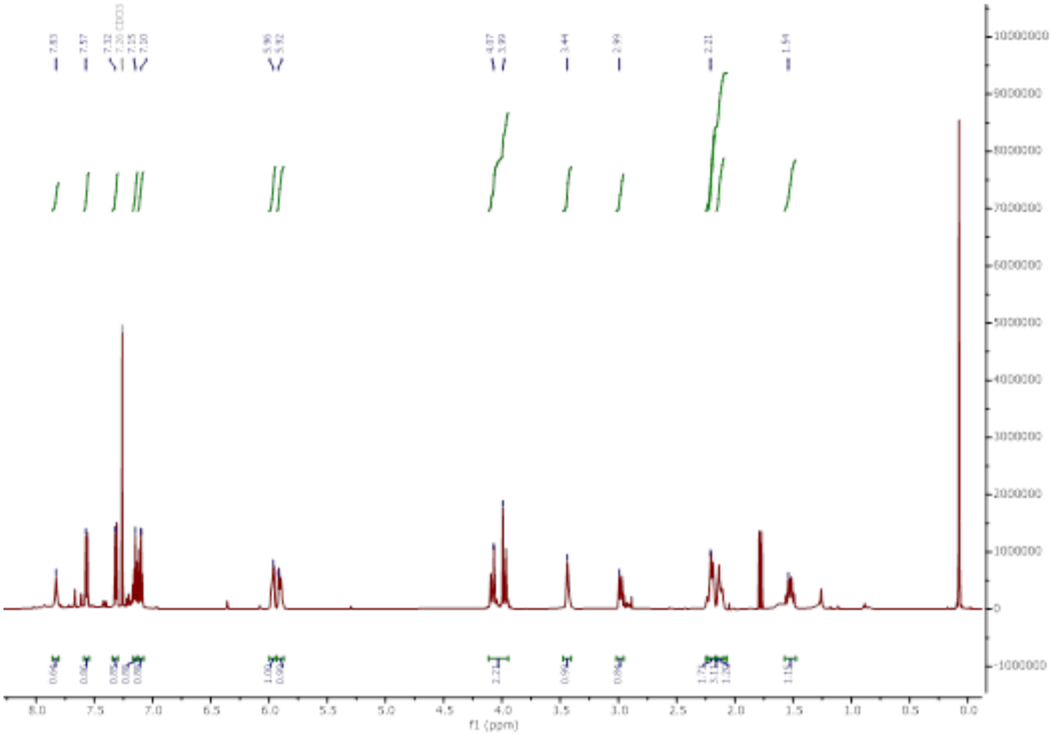

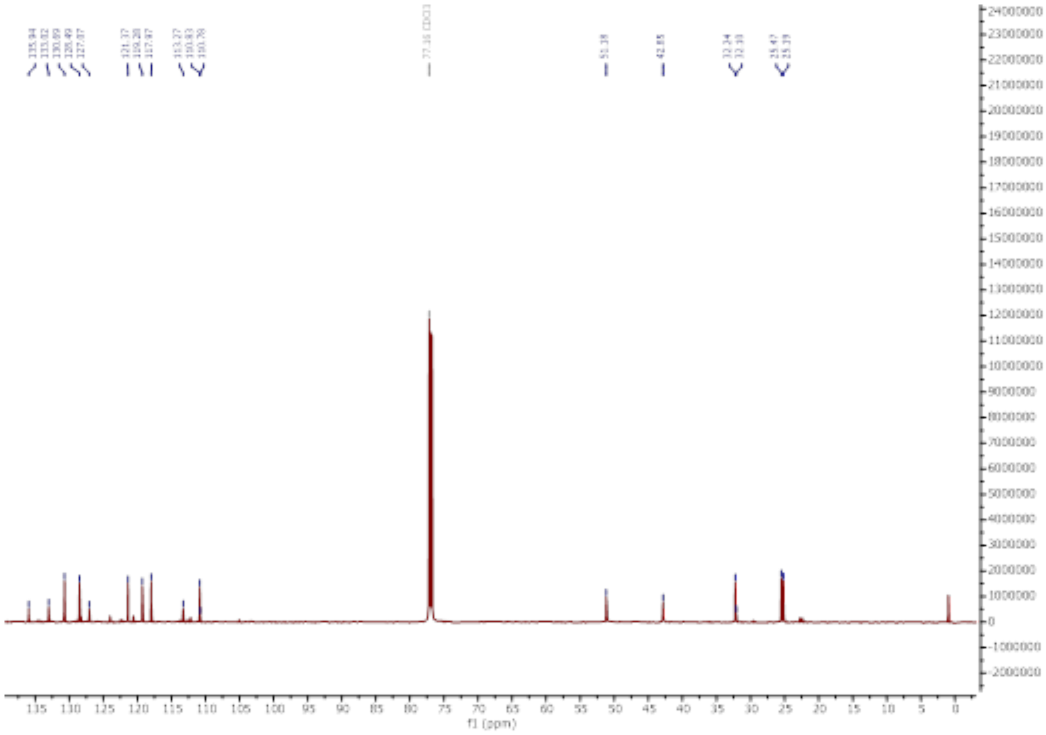

Figure S-15. Compound 26P ^{1}H NMR (800 MHz, CD_3CN, δ, 25 °C) & ^{13}C NMR (151 MHz, $CDCl_3$, δ, 25 °C)

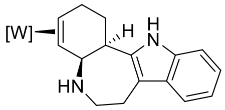

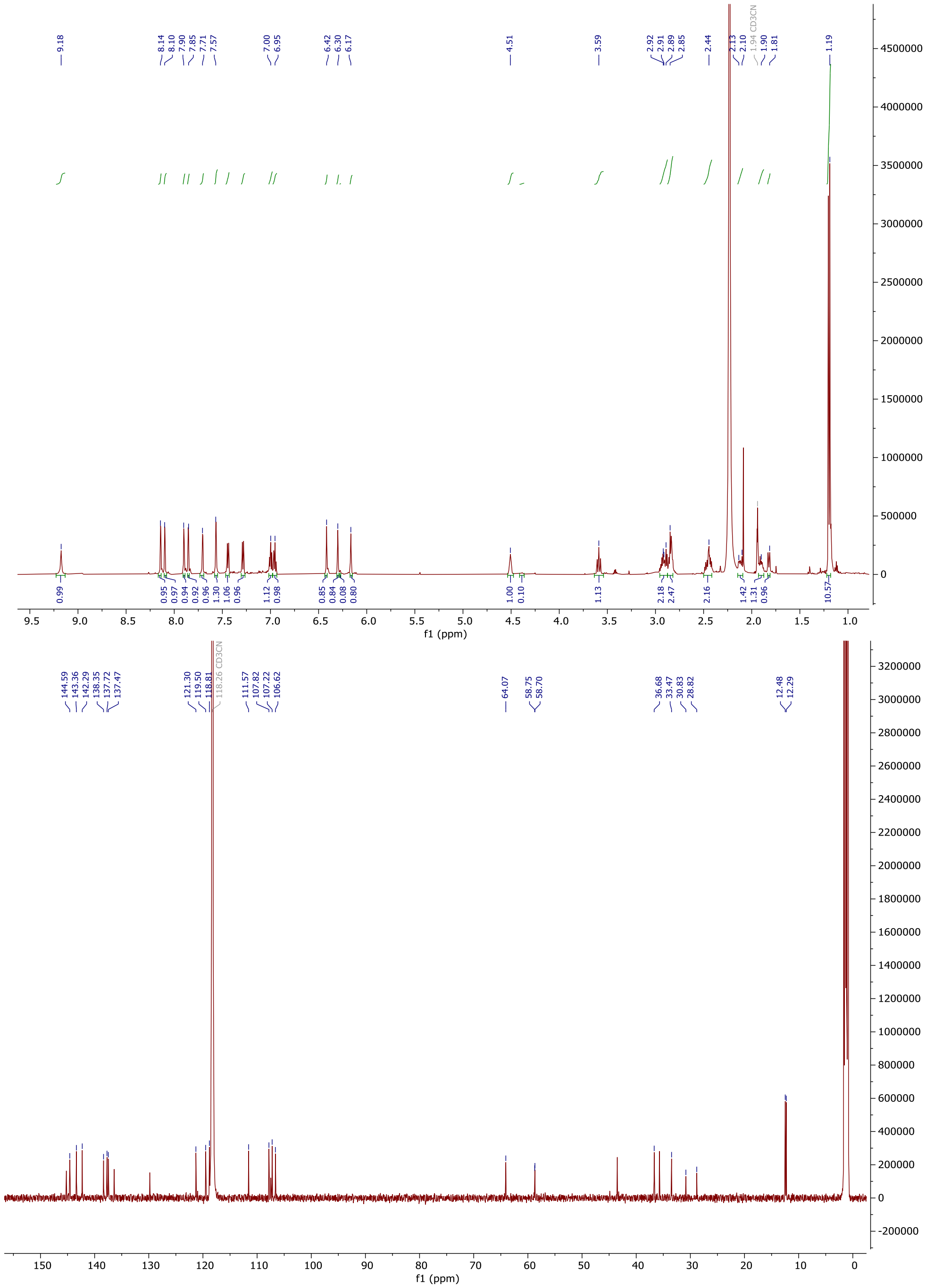

Figure S-16. Compound 27 ^{1}H NMR (800 MHz, CD_2Cl_2, δ, 25 °C) & ^{13}C NMR (201 MHz, CD_2Cl_2, δ, 25 °C).

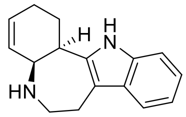

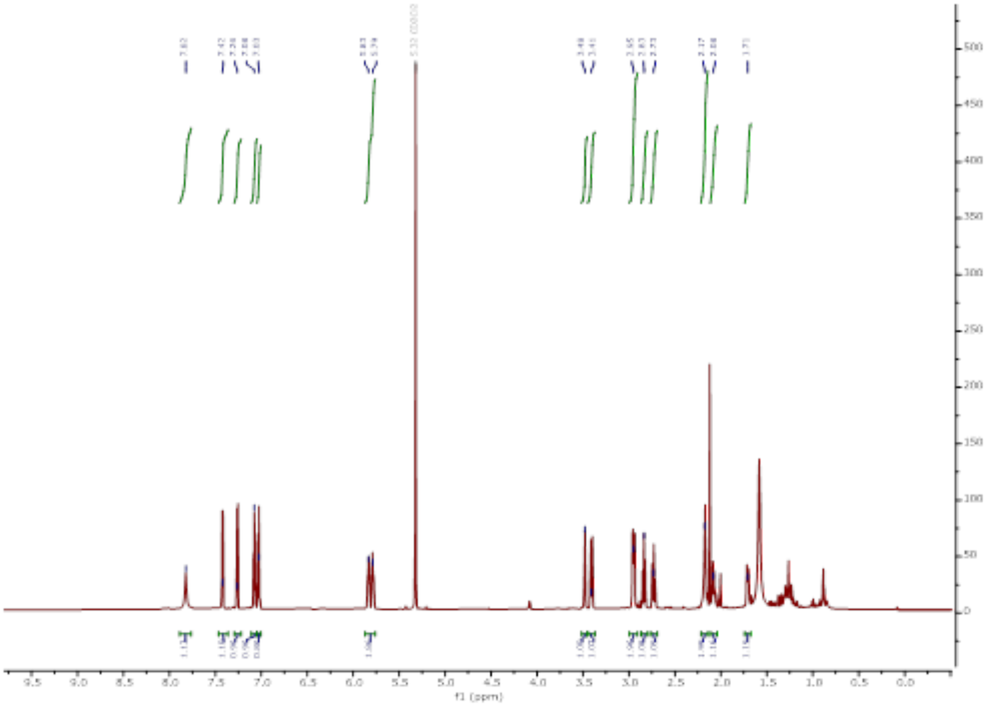

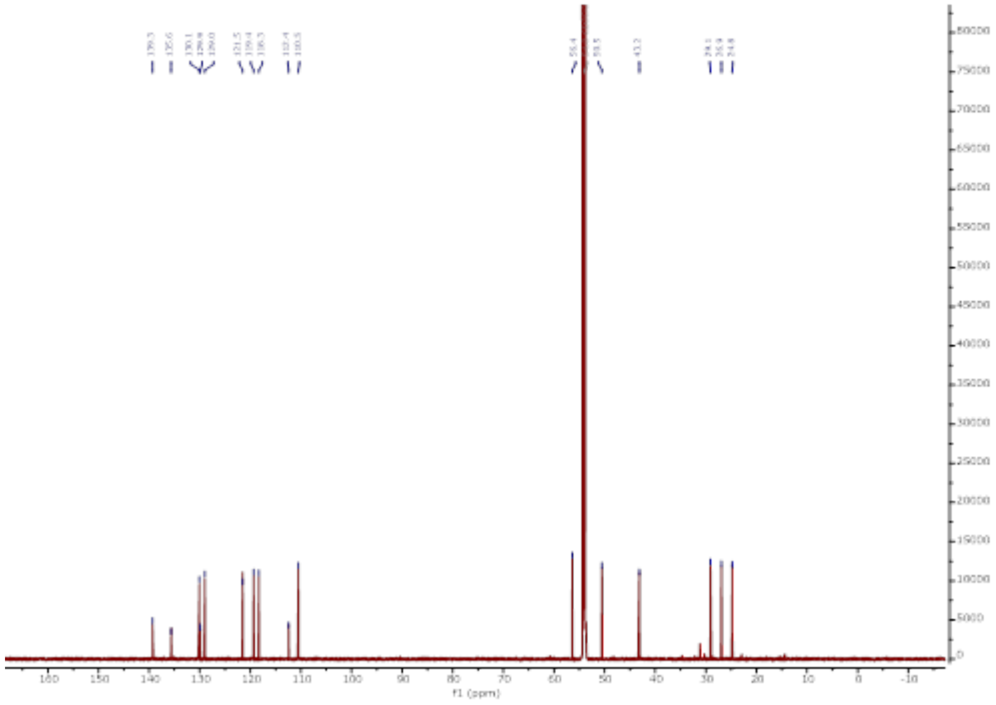

Crystallographic Data

A single crystal of each molecule listed in table S1 was coated with Paratone oil and mounted on a MiTeGen MicroLoop. The X-ray intensity data for all crystals were measured on a Bruker D8 Venture dual wavelength Mo/Cu Kappa four-circle diffractometer equipped with a PHOTON III detector and an Oxford Cryostream 800 or 800Plus low-temperature device. An Incoatec IμS 3.0 micro-focus sealed X-ray tube (Mo K_{α}, λ = 0.71073 Å) and a HELIOS double bounce multilayer mirror monochromator for were used for all crystals except for **26P**, which was collected on an Incoatec IμS 3.0 microfocus sealed X-ray tube (Cu K_{α}, λ=1.54178 Å) and HELIOS EF double bounce multilayer mirror monochromator.

All frames were integrated with the Bruker SAINT software package [1] using a narrow-frame algorithm. Data were corrected for absorption effects using the Multi-Scan method (SADABS) [2]. Each structure was solved using the Bruker SHELXT Software Package [3] within either APEX5 or APEX6 [1] or OLEX2. [4]

For all structures, non-hydrogen atoms were refined anisotropically using SHELXL. [5] The B-H hydrogen atom in each complex except **18** and **26P** was located in the electron density map and freely refined, as were the hydrogen atoms on the carbons bound directly to W in **10**, **14**, and **17**, and the O-H hydrogens in **9**. All other hydrogen atoms were placed in geometrically calculated positions with U_{iso} = 1.2U_{equiv} of the parent atom (U_{iso} = 1.5U_{equiv} for methyl). Most CIF files were prepared for publication using FinalCif.[6] Additional refinement details are given below.

The relative occupancy of the disordered atoms in **17** and **18** was freely refined, using constraints and/or restraints on the some of the disordered atoms and bonds. In **10** and **15**, there was severely disordered co-crystallized solvent that could not be adequately modeled with or without constraints. These solvents were accounted for using Platon SQUEEZE.[7] In **10**, void space of 840 Å^3 containing 283 electrons was found. This corresponds to a mixture of dichloromethane and pentane. In **15**, a void space of 77 Å^3 containing 27 electrons was found. We believe this corresponds to water and residual electron density from imperfections in the crystal (see vrf explanation embedded in CIF).

Table S-1. Crystal data for **10**, **14**, **15**, **17**, **18**, and **26P**.

	**10**	**14**	**15**	**17**	**18**	**26P**
CCDC number	2487622	2487623	2487624	2487625	2487626	2487627
Empirical formula	$C_{26}H_{34}BF_3N_7O_5PSW$	$C_{25}H_{33}BN_7O_2PW$	$C_{27}H_{36}BN_7O_5PW$	$C_{38}H_{53}BN_7O_4PW$	$C_{36}H_{49}BN_7O_3PW$	$C_{28}H_{37}BN_9OPW$
Formula weight	839.29	689.21	764.26	897.50	853.45	741.29
Temperature [K]	100.00	110.00	100.00	100.00	100.00	100.00
Crystal system	monoclinic	monoclinic	triclinic	monoclinic	monoclinic	triclinic
Space group (number)	P 2_1/c	P 2_1/n	P -1	P 2_1/c	P 2_1/c	P -1
a [Å]	13.3605(11)	10.1032(4)	8.1404(7)	14.7270(8)	10.0275(7)	10.2067(9)
b [Å]	10.2387(6)	20.1627(7)	10.1230(10)	16.0385(8)	24.2757(17)	12.922(2)
c [Å]	27.287(2)	13.0003(4)	20.1452(19)	17.6794(8)	15.6170(12)	13.363(2)
α [°]	90	90	78.544(3)	90	90	111.032(12)
β [°]	96.683(2)	94.0120(10)	79.061(3)	108.368(2)	107.718(2)	100.604(11)
γ [°]	90	90	71.338(3)	90	90	106.066(9)
Volume [Å^3]	3707.4(5)	2641.77(16)	1527.2(2)	3963.1(3)	3621.2(5)	1499.9(4)
Z	4	4	2	4	4	2
ρ_{calc} [gcm^{-3}]	1.504	1.733	1.662	1.504	1.565	1.641
μ [mm^{-1}]	3.272	4.471	3.883	3.003	3.280	7.951
F(000)	1664	1368	762	1824	1728	740

Crystal size [mm^3]	0.018 × 0.033 × 0.12	0.052 × 0.084 × 0.133	0.071 × 0.092 × 0.149	0.056 × 0.068 × 0.09	0.034 × 0.06 × 0.084	0.083 × 0.092 × 0.14
Crystal colour	colourless	colourless	colourless	colourless	colourless	colourless
Crystal shape	needle	plate	block	plate	Blocks	block
Radiation	Mo K_α (λ=0.71073 Å)	Mo K_α (λ=0.71073 Å)	Mo K_α (λ=0.71073 Å)	Mo K_α (λ=0.71073 Å)	Mo K_α (λ=0.71073 Å)	Cu K_α (λ=1.54178 Å)
2θ range [°]	4.04 to 51.41 (0.82 Å)	4.04 to 56.58 (0.75 Å)	4.29 to 52.77 (0.80 Å)	3.87 to 59.24 (0.72 Å)	4.26 to 50.82 (0.83 Å)	7.47 to 138.15 (0.83 Å)
Index ranges	$-16 \le h \le 16$<br>$-10 \le k \le 12$<br>$-33 \le l \le 33$	$-13 \le h \le 13$<br>$-26 \le k \le 26$<br>$-17 \le l \le 15$	$-10 \le h \le 10$<br>$-12 \le k \le 12$<br>$-25 \le l \le 25$	$-20 \le h \le 20$<br>$-22 \le k \le 19$<br>$-24 \le l \le 24$	$-12 \le h \le 12$<br>$-29 \le k \le 25$<br>$-18 \le l \le 17$	$-12 \le h \le 12$<br>$-15 \le k \le 15$<br>$-16 \le l \le 15$
Reflections collected	52442	45312	46014	77255	44566	18547
Independent reflections	7073<br>R_{int} = 0.1209<br>R_{sigma} = 0.0710	6555<br>R_{int} = 0.0504<br>R_{sigma} = 0.0319	6242<br>R_{int} = 0.0672<br>R_{sigma} = 0.0365	11125<br>R_{int} = 0.0713<br>R_{sigma} = 0.0490	6635<br>R_{int} = 0.1150<br>R_{sigma} = 0.0722	5445<br>R_{int} = 0.1521<br>R_{sigma} = 0.1217
Completeness to θ = 25.242°	100.0	100.0	99.9	100.0	99.9	99.3
Data / Restraints / Parameters	7073 / 0 / 429	6555 / 0 / 350	6242 / 0 / 387	11125 / 84 / 505	6635 / 74 / 402	5445 / 389 / 372
Goodness-of-fit on F^2	1.012	1.065	1.183	1.065	1.151	1.190
Final R indexes [$I \geq 2\sigma(I)$]	R_1 = 0.0383<br>wR_2 = 0.0750	R_1 = 0.0250<br>wR_2 = 0.0531	R_1 = 0.0645<br>wR_2 = 0.1576	R_1 = 0.0386<br>wR_2 = 0.0641	R_1 = 0.0682<br>wR_2 = 0.1615	R_1 = 0.1338<br>wR_2 = 0.3210
Final R indexes [all data]	R_1 = 0.0644<br>wR_2 = 0.0823	R_1 = 0.0315<br>wR_2 = 0.0557	R_1 = 0.0716<br>wR_2 = 0.1620	R_1 = 0.0561<br>wR_2 = 0.0689	R_1 = 0.1018<br>wR_2 = 0.1767	R_1 = 0.1901<br>wR_2 = 0.3757
Largest peak/hole [eÅ^{-3}]	1.09/−0.74	1.38/−0.55	3.59/−3.26	0.95/−1.80	2.54/−0.89	4.82/−1.02

Experimental Details for SI(1) to SI(4)

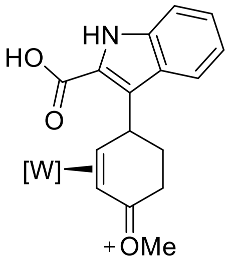

SI(1)

SI(1) has previously been communicated. [8]

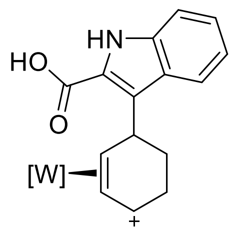

SI(2)

Used General Procedure **2** with **SI(1)** (0.5590 g, 0.6061 mmol), $NaBH_4$ (0.3375 g, 8.921 mmol), and HOTf (0.2982 g, 1.987 mmol) in DME (3 mL). Tan solid (0.4898 g, (91%)).

^{1}H NMR (600 MHz, CD_3CN) δ: 9.88 (s, 1H), 8.44 (d, *J* = 2.3 Hz, 1H), 8.19 (d, *J* = 2.3 Hz, 1H), 8.13 (d, *J* = 8.2 Hz, 1H), 8.03 – 7.98 (m, 1H), 7.95 (dt, *J* = 2.4, 0.8 Hz, 1H), 7.83 (dt, *J* = 2.6, 0.8 Hz, 1H), 7.79 (d, *J* = 2.4 Hz, 1H), 7.55 (dt, *J* = 8.4, 1.0 Hz, 1H), 7.38 (ddd, *J* = 8.2, 6.9, 1.1 Hz, 1H), 7.20 (ddd, *J* = 8.1, 6.9, 1.0 Hz, 1H), 6.59 (t, *J* = 7.1 Hz, 1H), 6.52 (t, *J* = 2.3 Hz, 1H), 6.46 (t, *J* = 2.3 Hz, 1H), 6.36 (t, *J* = 2.4 Hz, 1H), 5.57 – 5.47 (m, 1H), 5.13 (t, *J* = 8.9 Hz, 1H), 4.60 (dd, *J* = 15.6, 7.1 Hz, 1H), 3.61 (ddd, *J* = 18.8, 11.5, 6.4 Hz, 1H), 3.40 – 3.34 (m, 1H), 1.77 – 1.69 (m, 1H), 1.67 – 1.59 (m, 1H), 1.00 (d, *J* = 10.0 Hz, 9H). **^{13}C NMR (151 MHz, CD_3CN) δ**: 162.94, 149.26, 146.18, 143.19, 139.60, 139.55, 139.46, 137.89, 133.98, 129.86, 126.46, 126.20, 123.45, 123.31, 121.09, 113.71, 109.46, 109.00, 108.10, 105.88, 75.88 (d, *J* = 12.3 Hz), 35.44 (d, *J* = 3.2 Hz), 28.57, 26.09, 13.49 (d, *J* = 32.7 Hz) **ESI-HRMS (m/z):** $[M]^+$ calculated for $C_{27}H_{33}BN_8O_3PW^+$ 743.2010, found **743.2013**

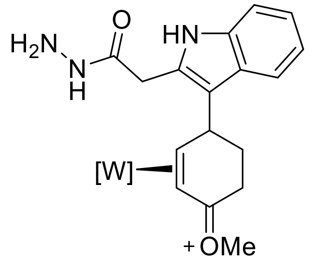

SI(3)

To a medium test-tube charged with a stir-pea was added (1) (106 mg, 0.139 mmol) and MeCN (1 mL). This test-tube was chilled to -30 °C for five min. To a separate medium test-tube charged with a stir-pea was added 2-(1*H*-indol-3-yl)acetohydrazide (156 mg, 0.824 mmol) and MeCN (1 mL), and this test-tube

was also chilled for five min. In the third test tube was added MeCN (1 mL) and HOTf (131 mg, 0.873 mmol). After chilling this solution of HOTf to -30 °C for five min, the solution was transferred to the solution of (1), and the resulting mixture was allowed to stir at -30 °C for five min. A fourth test-tube containing MeCN (1 mL) and HOTf (167 mg, 1.11 mmol) was then prepared and chilled to -30 °C for five min. This solution of HOTf was then transferred to the solution of 2-(1*H*-indol-3-yl)acetohydrazide and the resulting mixture was allowed to stir at -30 °C for five min, after which it was transferred to the solution of (1).The reaction was allowed to proceed at -30 °C for 21 h, after which it removed from the cold bath, diluted with DCM (5 mL), and washed with DI water (3 x 3 mL). The organic layer was dried over anhydrous Na_2SO4, which was subsequently removed by filtering the organic layer through a 15 mL fine-porosity fritted disc. The filtrate was then concentrated *in vacuo* to a brown oil, which was then redissolved in DCM (minimal) and precipitated into stirring Et_2O (200 mL). The resulting precipitate was collected on a 15 mL fine-porosity fritted disc and desiccated under static vacuum to provide S(1) (89 mg, 0.094 mmol, 67% yield). **^{1}H NMR (800 MHz, CD_3CN) δ:** 9.58 (s, 1H), 8.20 (d, J = 2.4 Hz, 1H), 8.09 (d, J = 2.3 Hz, 1H), 8.03 (d, J = 2.3 Hz, 1H), 7.96 (d, J = 2.4 Hz, 1H), 7.94 (d, J = 2.4 Hz, 1H), 7.52 (m, 2H),* 7.42 (d, J = 8.1 Hz, 1H), 7.14 (t, J = 7.6 Hz, 1H), 7.07 (t, J = 7.5 Hz, 1H), 6.52 (t, J = 2.3 Hz, 1H), 6.48 (t, J = 2.3 Hz, 1H), 6.39 (t, J = 2.4 Hz, 1H), 4.63 (m, 2H),* 3.74 (d, J = 15.9 Hz, 1H), 3.64 (d, J = 15.9 Hz, 1H), 3.37 (d, J = 8.0 Hz, 1H), 3.10 (m, 4H),* 2.78 (m, 1H), 2.33 (m, 1H), 1.90 (m, 1H), 1.07 (d, J = 9.6 Hz, 9H). **^{13}C NMR (201 MHz, CD_3CN) δ:** 196.4, 172.3, 146.0, 145.0, 143.9, 143.2, 140.1, 139.9, 139.4, 136.5, 129.9, 122.5, 120.4, 119.2, 112.1, 109.1, 108.9, 108.7, 105.2, 73.6 (d, J_{CP} = 15.1 Hz), 66.4, 58.7, 36.9 (d, J_{CP} = 2.6 Hz), 33.1, 31.0, 30.0, 14.0 (d, J_{CP} = 31.8 Hz). **CV** (MeCN; 100 mV/s): $E_{p,c}$ = -1.28 V (NHE). **APCI-HRMS** (m/z): [M]+ calculated for $[C_{29}H_{39}BN_{10}O_3PW]^+$: 801.2541, found 801.2541. **IR**: ν(NH) = 3309 cm^{-1}, ν(CO) = 1612 cm^{-1}

*Two overlapping proton signals as indicated by HSQC.

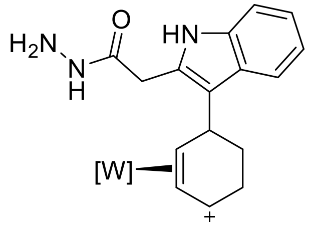

SI(4)

Used General Procedure 1 with **S(3)** (726 mg, 0.764 mmol), $NaBH_4$ (142 mg, 3.75 mmol), and HOTf (200 mg, 1.33 mmol). Obtained **S(2)** as a pink-tan powder (476 mg, 0.517 mmol, 68% yield). **^{1}H NMR (800 MHz, CD_3CN) δ:** 8.42 (br. s, 1H), 8.15 (br. s, 1H), 8.05 (br. s, 1H), 8.02 (d, *J* = 2.5 Hz, 1H), 7.97 (d, *J* = 2.5 Hz, 1H), 7.83 (d, *J* = 2.6 Hz, 1H), 7.50 (d, *J* = 8.3 Hz, 1H), 7.45 (d, *J* = 8.1 Hz, 1H), 7.16 (t, *J* = 7.4 Hz, 1H), 7.09 (t, *J* = 7.5 Hz, 1H), 6.66 (t, *J* = 7.2 Hz, 1H), 6.54 (m, 2H),* 6.36 (t, *J* = 2.4 Hz, 1H), 5.43 (t, *J* = 7.5 Hz, 1H), 4.60 (dd, *J* = 7.1, 15.8 Hz, 1H), 4.07 (dd, *J* = 6.3, 11.1 Hz, 1H), 3.84 (d, *J* = 16.8 Hz, 1H), 3.75 (d, *J* = 16.8 Hz, 1H), 3.50 (m, 1H), 3.33 (m, 1H), 1.90 (m, 1H), 1.40 (m, 1H), 1.02 (d, *J* = 10.0 Hz, 9H). **^{13}C NMR (201 MHz, CD_3CN) δ:** 171.8, 149.5, 146.4, 143.6, 143.4, 139.7, 139.7, 139.7, 137.3, 136.8, 129.2, 122.9, 120.6, 119.2, 112.3, 109.7, 109.3, 108.2, 105.1, 103.1, 71.6 (d, J_{CP} = 12.8 Hz), 36.9, 30.3, 29.4, 25.9, 13.7 (d, J_{CP} = 32.7 Hz).

*Two overlapping proton signals as indicated by HSQC.

Figure S-17. Compound SI(2) ^{1}H NMR (600 MHz, CD_3CN, δ, 25 °C) & ^{13}C NMR (151 MHz, CD_3CN, δ, 25 °C)

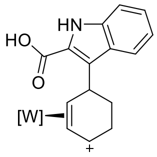

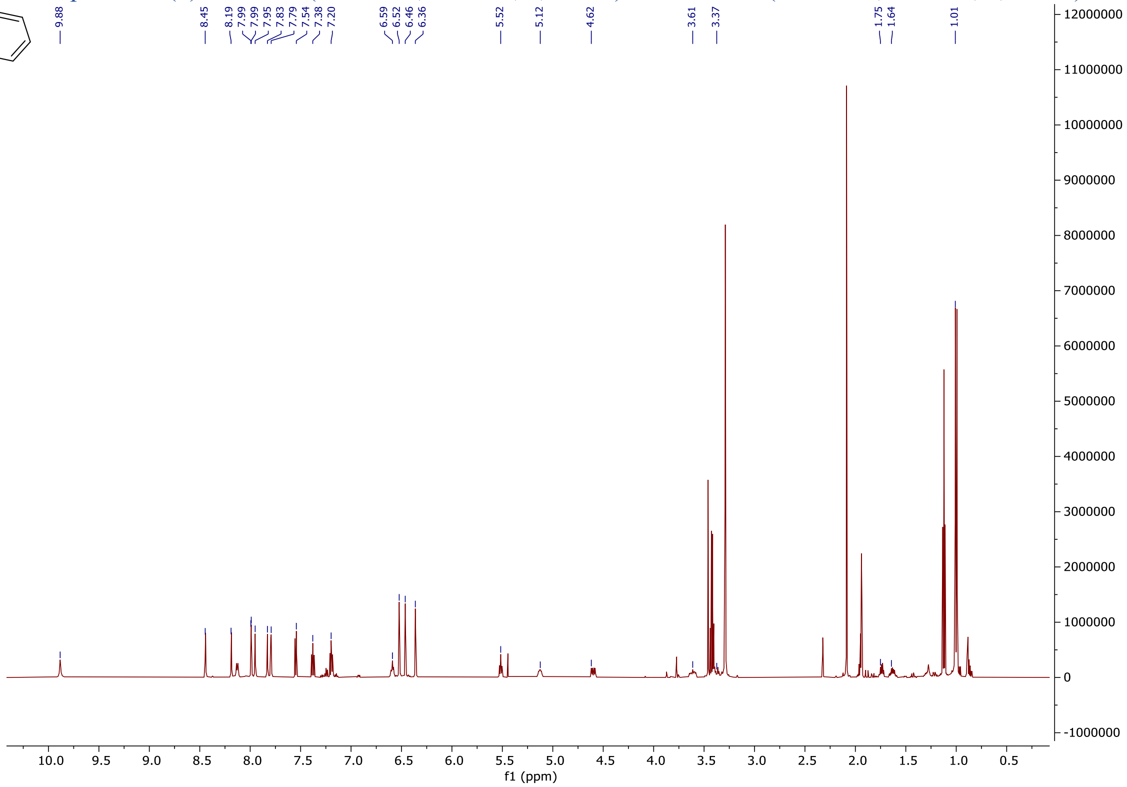

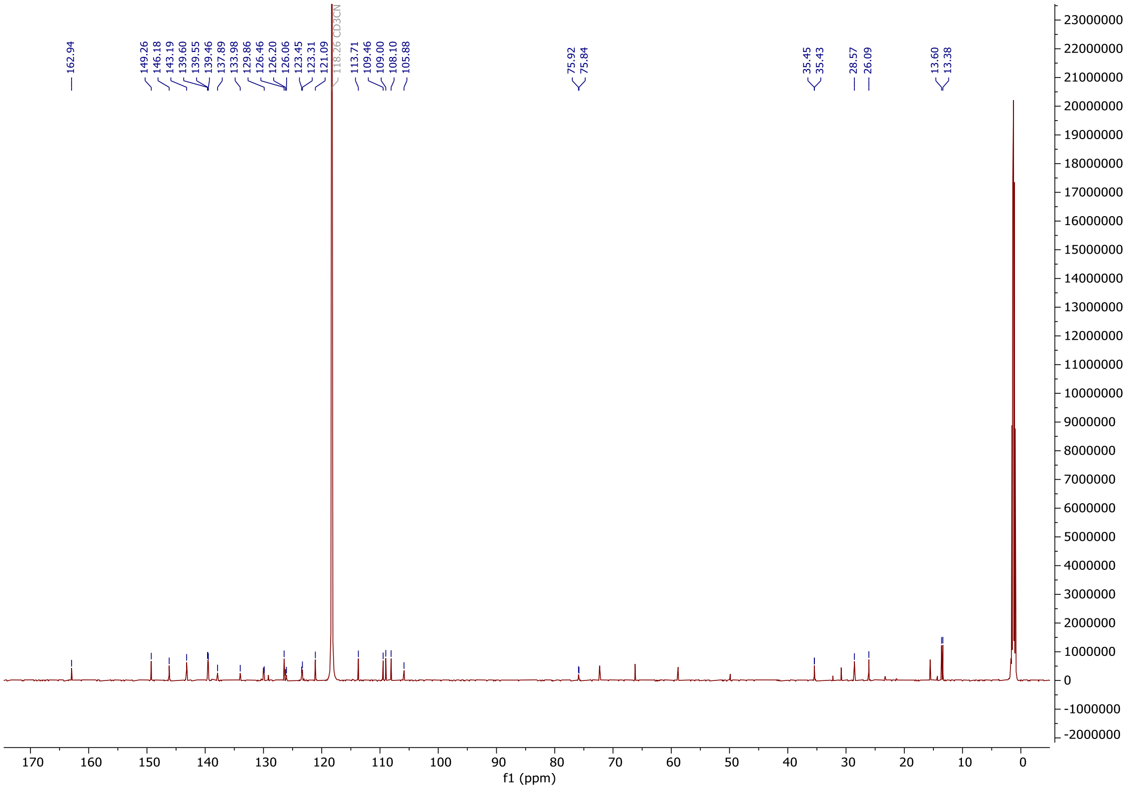

Figure S-18. Compound SI(3) ^{1}H NMR (800 MHz, CD_3CN, δ, 25 °C) & ^{13}C NMR (201 MHz, CD_3CN, δ, 25 °C).

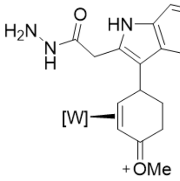

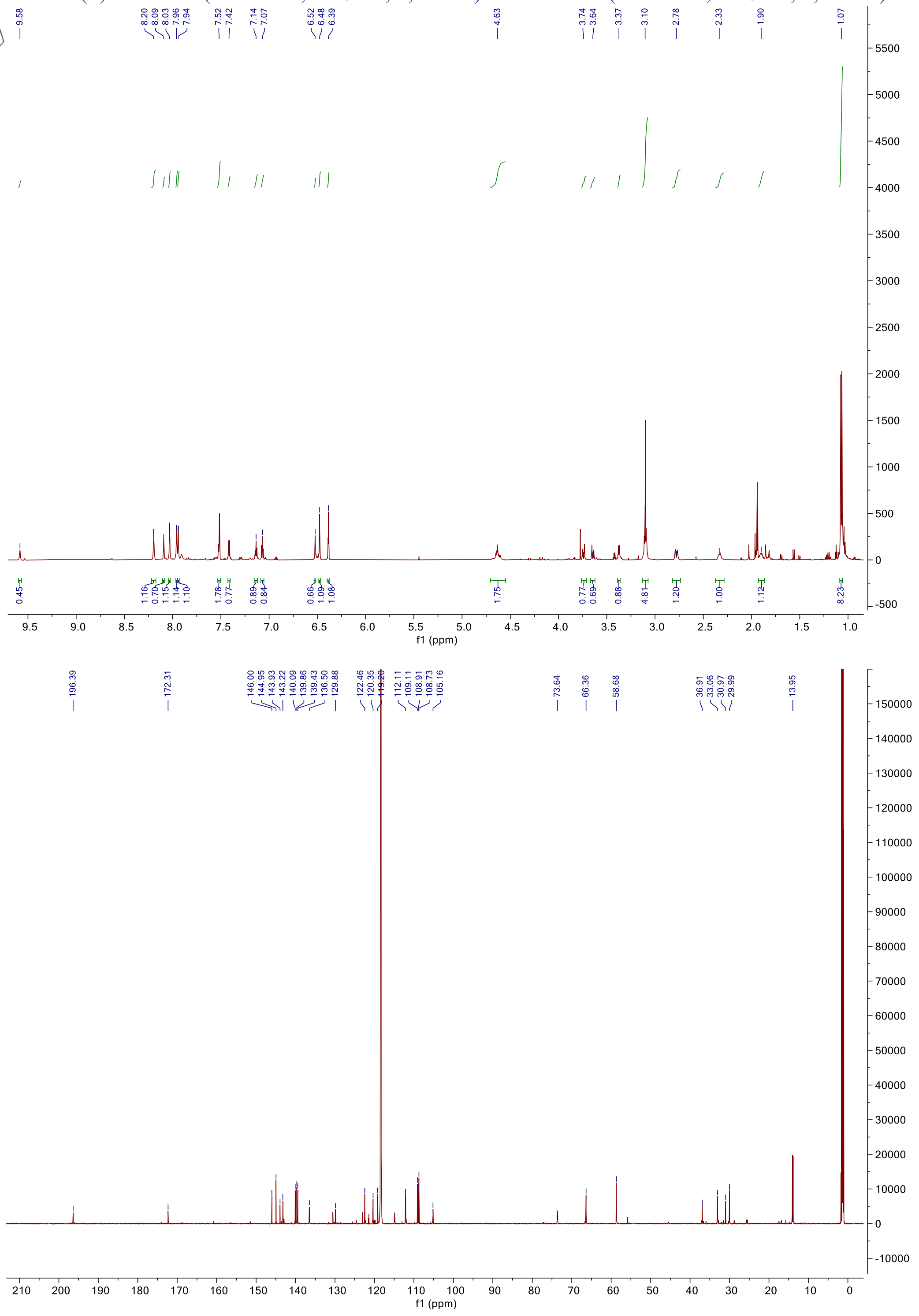

Figure S-19. Compound SI(4) ^{1}H NMR (800 MHz, CD_3CN, δ, 25 °C) & ^{13}C NMR (201 MHz, CD_3CN, δ, 25 °C).

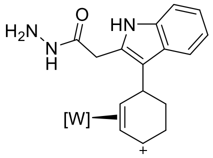

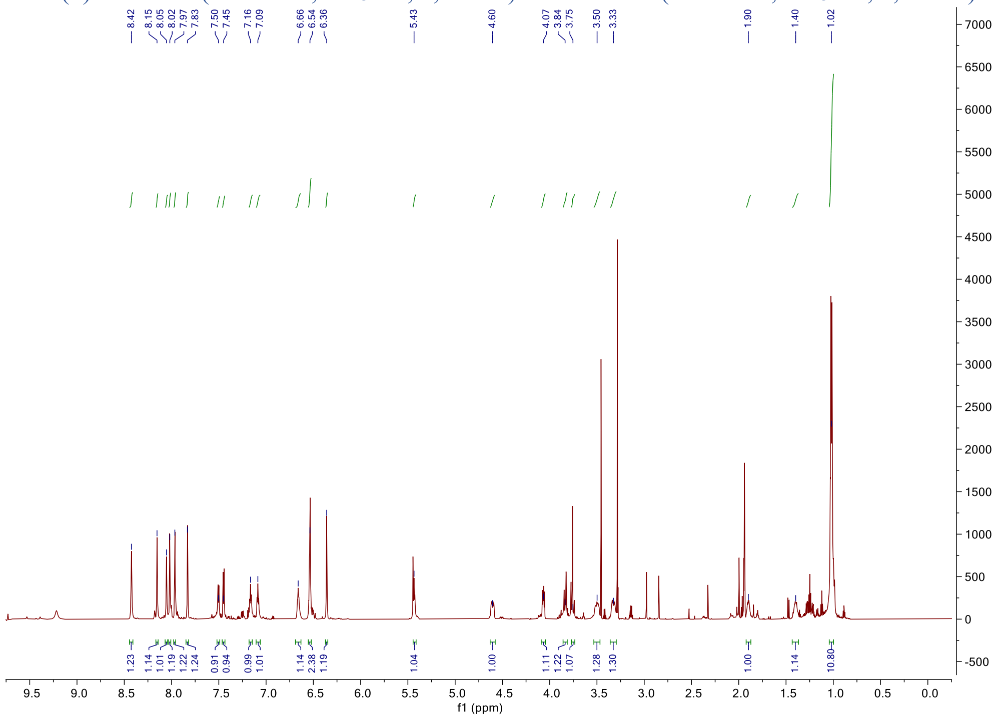

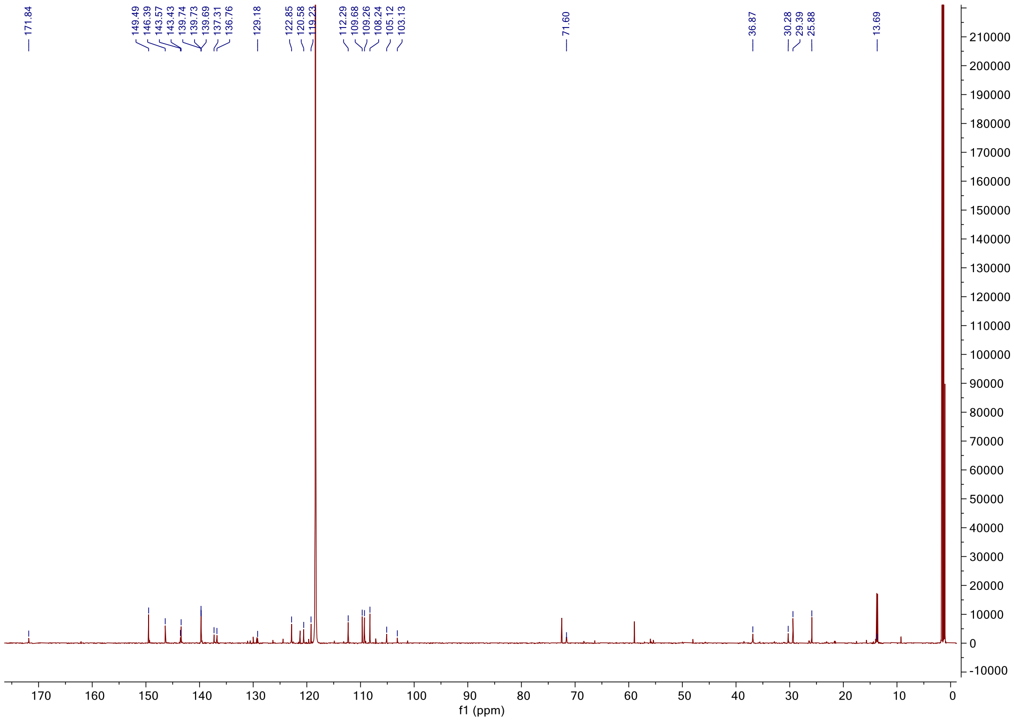

Supplementary References

1. Bruker (2019). *Saint; APEX3, APEX4, APEX5, APEX6*. Bruker AXS Inc., Madison, Wisconsin, USA.
2. Krause, L.; Herbst-Irmer, R; Sheldrick, G. M.; Stalke, D., Comparison of silver and molybdenum microfocus X-ray sources for single-crystal structure determination. *J. Appl. Cryst.* **2015**, *48*, 3-10. doi:10.1107/ S1600576714022985
3. Sheldrick, G. M., SHELXT – Integrated space-group and crystal structure determination. *Acta Crystallogr. Sect. A: Found. Adv.* **2015**, *A71*, 3-8. doi:10.1107/S2053273314026370
4. Dolomanov, O. V.; Bourhis, L. J.; Gildea, R. J.; Howard, J. A. K.; Puschmann, H., OLEX2: a complete structure solution, refinement and analysis program. *J. Appl. Cryst.* **2009**, ***42***, 339-341. doi:10.1107/S0021889808042726
5. Sheldrick, G. M., Crystal structure refinement with SHELXL. *Acta Crystallogr. Sect C: Struct. Chem.* **2015**, *C71*, 3-8. doi: 10.1107/S2053229614024218
6. Kratzert, D. FinalCif, https://dkratzert.de/finalcif.html.
7. Spek, A. L., PLATON SQUEEZE: a tool for the calculation of the disordered solvent contribution to the calculated structure factors. *Acta Crystallogr. Sect C: Struct. Chem.* **2015**, *C71*, 9-18. doi: 10.1107/S2053229614024929
8. Weatherford-Pratt, J. T.; Bloch, J. M.; Smith, J. A.; Ericson, M. N.; Siela, D. J.; Ortiz, M. R.; Shingler, M. H.; Fong, S.; Laredo, J. A.; Patel, I. U.; McGraw, M.; Dickie, D. A.; Harman, W. D., Tungsten-anisole complex provides 3,6-substituted cyclohexenes for highly diversified chemical libraries. *Science Advances* **2024,** *10* (7), eadl0885.
